# The Heterotrimeric Transcription Factor CCAAT-Binding Complex and Ca^2+^-CrzA Signaling Reversely Regulate the Transition between Fungal Hyphal Growth and Asexual Reproduction

**DOI:** 10.1128/mBio.03007-21

**Published:** 2021-11-16

**Authors:** Yiran Ren, Chi Zhang, Ziqing Chen, Ling Lu

**Affiliations:** a Jiangsu Key Laboratory for Microbes and Functional Genomics, Jiangsu Engineering and Technology Research Centre for Microbiology; College of Life Sciences, Nanjing Normal University, Nanjing, China; Karlsruhe Institute of Technology (KIT)

**Keywords:** submerged conidiation, CCAAT-binding complex, calcium, CrzA, *Aspergillus fumigatus*

## Abstract

The life cycle of filamentous fungi generally comprises hyphal growth and asexual reproduction. Both growth and propagation processes are critical for invasion growth, spore dissemination, and virulence in fungal pathogens and for the production of secondary metabolites or for biomass accumulation in industrial filamentous fungi. The CCAAT-binding complex (CBC) is a heterotrimeric transcription factor comprising three subunits, HapB, HapC, and HapE, and is highly conserved in fungi. Previous studies revealed that CBC regulates sterol metabolism by repressing several genes in the ergosterol biosynthetic pathway in the human fungal pathogen Aspergillus fumigatus. In the present study, we found dysfunction of CBC caused the abnormal asexual reproduction (conidiation) in submerged liquid culture. CBC suppresses the activation of the *brlA* gene in the central regulatory pathway for conidiation combined with its upstream regulators *fluG*, *flbD*, and *flbC* by binding to the 5′-CCAAT-3′ motif within conidiation gene promoters, and lack of CBC member HapB results in the upregulation of these genes. Furthermore, when the expression of *brlA* or *flbC* is repressed, the submerged conidiation does not happen in the *hapB* mutant. Interestingly, deletion of HapB leads to enhanced transient cytosolic Ca^2+^ levels and activates conidiation-positive inducer Ca^2+^-CrzA modules to enhance submerged conidiation, demonstrating that CrzA works with CBC as a reverse regulator of fungal conidiation. To the best of our knowledge, the finding of this study is the first report for the molecular switch mechanism between vegetative hyphal growth and asexual development regulated by CBC, in concert with Ca^2+^-CrzA signaling in A. fumigatus.

## INTRODUCTION

Aspergillus fumigatus is a common environmental saprobe mold and plays an important role in carbon and nitrogen recycling by decomposing dead organic biomass (1, 2). In addition, A. fumigatus is a leading cause of invasive mold infections in immunocompromised individuals (3–6). In the natural environment or on solid culture plates, A. fumigatus first grows in a vegetative state and is composed of long branched filamentous structures that are able to attach to the substrate upon which the fungus feeds. Under host conditions, a fungal colony is initiated from the germination of a single spore that gives rise to a network of hyphae referred to as invasive hyphal growth, which damages or destroys host organ tissue (6, 7). When vegetative hyphae encounter some unsuitable growth conditions or are exposed to air, aerial hyphae form asexual conidiophores associated with conidia and initiate an asexual reproductive cycle (8–11). A. fumigatus propagates asexually via spores that can be dispersed over large geographical distances in the air, and these spores can germinate to grow under a broad range of environmental conditions (1, 12–14). The genetic mechanisms of asexual reproduction (conidiation) have been extensively studied in the model fungus Aspergillus nidulans, and some have also been studied in A. fumigatus (8, 15, 16). In both species, the activation of *brlA* encoding a conserved C_2_H_2_ zinc finger transcription factor, BrlA (bristle), is a key event in response to the development induction signal (16–19). Previous studies have identified that *brlA* and its targets, *abaA* and *wetA* (BrlA→AbaA→WetA), comprise a central regulatory pathway that controls the spatial and temporal expression of conidiation-specific genes during conidiogenesis (20). In addition to its involvement in this central regulatory pathway, later studies identified that *brlA* expression could be affected by several upstream developmental activators, *fluG*, *flbA*, *flbB*, *flbC*, *flbD*, and *flbE*, and mutations in these genes result in “fluffy” colonies with downregulated *brlA* (8, 15, 16, 21–25). Moreover, through combinatorial genetic studies, SfgA, VosA, VeA, VelB, VelC, NsdC, and NsdD, which repress the expression of *brlA* as negative regulators of conidiation, have been found (26–30). Notably, current knowledge of conidiation-related regulators in Aspergillus was mainly obtained from studies under solid-culture conditions. In comparison, submerged cultured hyphae in A. fumigatus and in all other filamentous fungi undergo constant vegetative growth to form a large number of mycelial pellets, whereas asexual conidiation rarely occurs (27). In addition, during progress of the invasive aspergillosis, germinated spores display high-speed tip growth, and conidiation rarely occurs (17, 31). These phenomena suggest that Aspergillus species have the ability to finely sense the environment and decide when or where to undergo hyphal extension or initiate conidiation. Thus, the transition from vegetative growth to conidiation in Aspergillus species is a crucial process contributing to the long-term survival, propagation, and fitness of fungi in response to various environmental conditions (32, 33). To date, several reports have demonstrated that the loss of each of the velvet regulators VosA, VeA, and VelB; SfgA (the FluG suppressor); and NsdC and NsdD (the sexual reproduction activators) causes abnormal submerged hyperconidiation (26–28, 30, 34), suggesting that these regulators may exert negative influence on asexual conidiation in liquid-submerged culture. However, whether or how these genes work together with a known central regulatory pathway and its upstream developmental activators is still poorly understood.

The CCAAT-binding complex (CBC) is a heterotrimeric transcription factor comprising three subunits (HapB/HapC/HapE) that is involved in the regulation of sterol metabolism by repressing several genes in the ergosterol biosynthetic pathway (35). In the present study, we found a new function for CBC in A. fumigatus as a negative regulator of conidiation under liquid culture conditions. The lack of each member of the heterotrimeric CBC led to abnormal A. fumigatus conidiation in liquid-submerged culture, suggesting that CBC plays an important role in maintaining normal vegetative growth. As a transcription factor, CBC participates in numerous cellular processes, such as iron homeostasis, sterol biosynthesis, oxidative stress response, secondary metabolism, and development, by regulating the expression of related genes. However, the negative regulation of conidiation by CBC in liquid-submerged culture has not yet been reported. Here, we identified that CBC could directly bind to the CCAAT motif within the promoters of *brlA*, *fluG*, *flbD*, and *flbC*, and lack of CBC member-HapB caused the upregulation of these genes, suggesting that CBC suppresses the activation of the central regulatory pathway combined with its upstream regulators to inhibit conidiation in submerged culture. Notably, the submerged conidiation in the CBC mutants was markedly enhanced by the addition of calcium. In contrast, the calcium-chelating agent EGTA remarkably suppressed the abovementioned submerged conidiation. Calcium, a secondary messenger, plays important roles in regulating fungal growth, development, and reproduction. The transcription factor calcineurin-responsive zinc finger (CrzA) is a central regulator of Ca^2+^-mediated signals. Through a conditional *crzA* gene-Tet on/off system, we demonstrated that CrzA, as a positive conidiation inducer, works with CBC to control asexual reproduction in A. fumigatus in submerged culture.

## RESULTS

### Lack of CBC causes desuppression of A. fumigatus conidiation in liquid-submerged culture.

**FIG 1:**
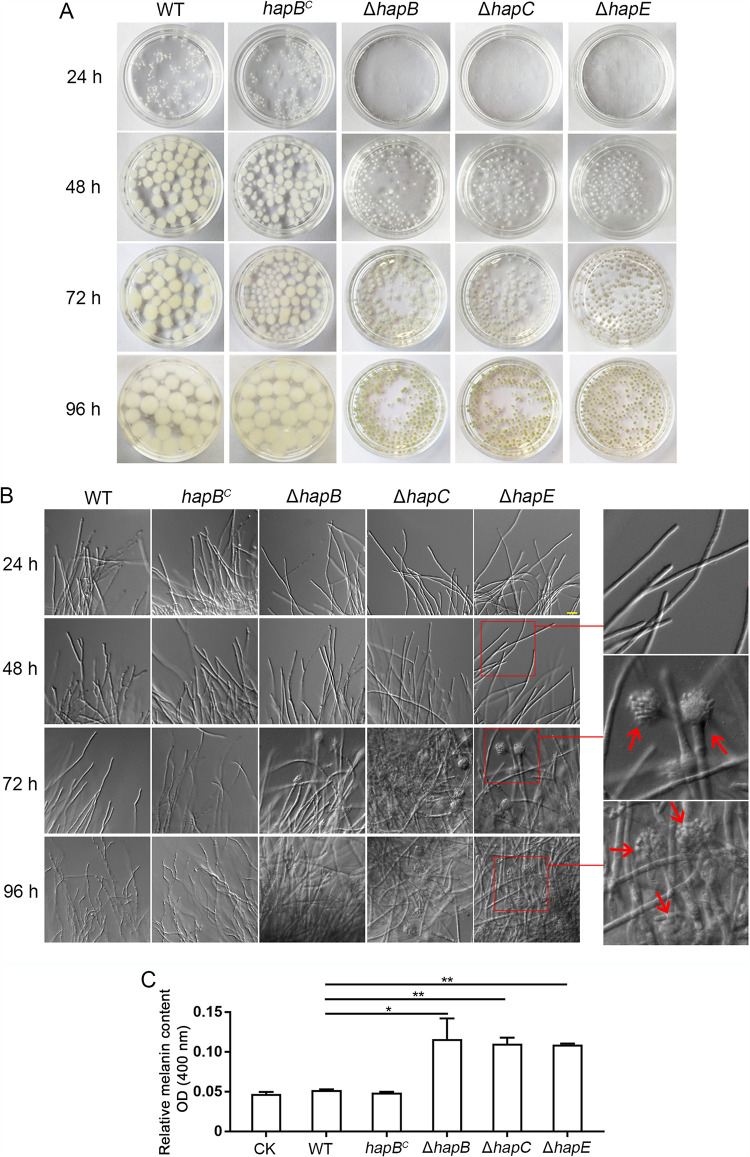
Phenotypes of the CBC mutants in liquid minimal medium (MM). (A and B) A total of 3.5 × 10^6^ spores were grown in liquid MM for the indicated days in a shaker at 37°C. The macromorphology and hyphae/asexual conidiogenous structure of mycelial pellets were observed. Scale bar represents 10 μm. (C) The OD_400_ value of the supernatant with dissolved melanin represented the relative melanin content. CK indicates the OD_400_ value of control check which was obtained by measuring the optical density of buffer extracted for melanin at 400 nm. Statistical significance was determined by Student’s *t* test. *, *P < *0.05; **, *P < *0.01.

Generally, conidiation initiation is suppressed in A. fumigatus under liquid shaking culture conditions. As shown in Fig. 1A and B, the parental wild-type (WT) strain displayed constant hyphal vegetative growth to form many colorless mycelial pellets. Interestingly, under the same culture conditions, the CBC mutants (Δ*hapB*, Δ*hapC*, and Δ*hapE*) showed pale-green mycelial pellets presenting conidiophores that popped out, suggesting the occurrence of asexual conidiation in a process referred to as submerged conidiation. Furthermore, the mycelial pellets of the CBC mutants were significantly smaller and exhibited less biomass than those of the WT strain or *hapB*-complemented strain (*hapB^C^*) (Fig. 1A and see Fig. S1A in the supplemental material). Generally, asexual conidiation is accompanied by melanin accumulation on the conidial surface in Aspergillus species (36–38). Next, we extracted the melanin with NaOH lysis buffer from mycelial pellets and found that the Δ*hapB*, Δ*hapC*, and Δ*hapE* mutants truly had highly accumulated melanin contents compared with the WT or the *hapB^C^* strain (Fig. 1C). It implies that CBC might be a suppressor of conidiation in liquid culture. However, on solid medium, the A. fumigatus CBC mutants displayed significantly fewer conidia than did the wild-type and the *hapB^C^* strains (Fig. S1B to D). These data suggest that CBC may have different functions for asexual development on solid medium than under liquid culture conditions.

10.1128/mBio.03007-21.1 FIG S1 (A) Spores (3.5 × 10^6^) were grown in liquid MM for the indicated days in a shaker at 37°C. The dry weight (biomass) of the resulting mycelial pellets was measured. (B to D) Spores (2 × 10^5^) were inoculated on solid MM at 37°C for the indicated days. Morphology, diameter, and conidial amount of the resulting colonies. Statistical significance was determined by Student’s *t* test. **, *P < *0.01. Download FIG S1, TIF file, 0.9 MB. Copyright © 2021 Ren et al. 2021 Ren et al. https://creativecommons.org/licenses/by/4.0/ This content is distributed under the terms of the Creative Commons Attribution 4.0 International license.

### Transcriptome analyses of the Δ*hapB* mutant reveal altered expression patterns for genes related to conidiation and pigment synthesis.

**FIG 2:**
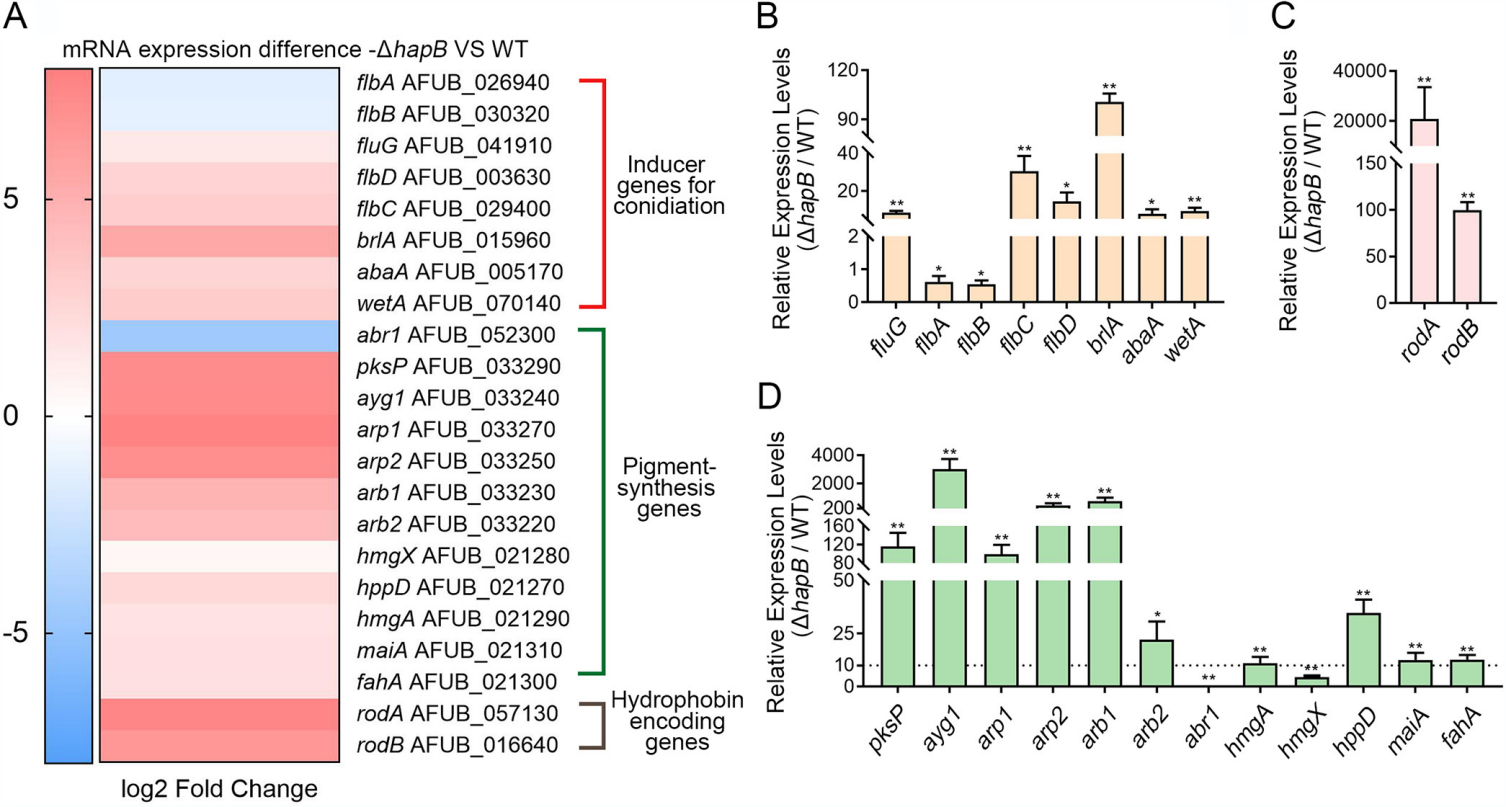
A comparison of gene expression related to conidiation and pigment synthesis in RNA-seq and RT-qPCR analyses for the Δ*hapB* mutant relative to the WT strain. (A) Heat map of RNA-seq data for the 22 selected genes. (B to D) The transcript levels of the indicated genes in WT and Δ*hapB* strains grown at 37°C for 48 h. Statistical significance was determined by Student’s *t* test. *, *P < *0.05; **, *P < *0.01; ns, not significant.

Given that CBC deficiency induces the abnormal submerged conidiation, we wondered whether CBC affects the expression of conidiation-regulated genes under the liquid culture condition. A transcriptome profiling experiment (RNA-seq) was carried out to identify genes differentially expressed between WT and Δ*hapB* strains grown in liquid minimal medium (MM). The results showed that 4,592 (46.3%) of the total predicted 9,911 A. fumigatus transcripts were differentially expressed in the transcriptome database, with 2,424 genes shown to be upregulated at least 2-fold in the Δ*hapB* mutant compared to the WT strain (Data Set S1). Based on the biological processes, Gene Ontology (GO) enrichment analysis was performed. Notably, asexual spore wall assembly and cell cycle arrest belonged to the top 10 biological processes of the upregulated genes (Fig. S2), suggesting the inhibition of hyphal vegetative growth and the activation of asexual conidiation in the Δ*hapB* mutant. Among these upregulated genes (Fig. 2A), all the key central conidiation regulators (*brlA*, *abaA*, and *wetA*) and their relative upstream genes (*fluG*, *flbC*, and *flbD*) were remarkably upregulated, but *flbA* and *flbB* were slightly downregulated, which was further verified by real-time quantitative PCR (RT-qPCR) (Fig. 2B). According to previous reports, these central and upstream regulators of conidiation are mainly responsible for positive regulation during the process of conidiation. In addition, we also analyzed the majority of the reported negative regulators of conidiation at the mRNA level and found that none of the tested reported negative regulators, VosA, VeA, VelB, MpkB, SfgA, NsdC, and NsdD, showed significantly decreased expression. Instead, they displayed slight upregulation or no changes in the Δ*hapB* mutant compared to the WT strain (Fig. S3A and B). These results suggested that the submerged conidiation caused by the *hapB* mutant was not due to changed expression of the known negative regulators of conidiation. Notably, the submerged conidiation of the Δ*hapB* mutant was mainly associated with the upregulation of *brlA-*related central conidiation regulators and their upstream fluffy genes. Activation of the asexual conidiation pathway induces the synthesis of pigment and conidium-superficial hydrophobin protein (36–40). Correspondingly, we found that most pigment synthesis-related key genes, including dihydroxynaphthalene (DHN)-melanin genes (*pksP*, *ayg1*, *arp1*, *arp2*, *arb1*, and *arb2*) and promelanin genes (*hmgA*, *hppD*, *maiA*, and *fahA*), and conidium-hydrophobin-encoding genes *rodA* and *rodB*, were significantly upregulated in the Δ*hapB* mutant compared to the WT strain (Fig. 2A, C, and D). RNA-seq showed that the top 10 biological processes of the downregulated genes were mainly mitochondrial electron transport and biotin biosynthesis required for vegetative growth (Fig. S2), corresponding to the phenotype of decreased hyphal biomass in the Δ*hapB* strain cultured in liquid MM (Fig. S1A). Taken together, our studies suggest that lack of the CBC subunit HapB derepresses conidiation by upregulating *brlA*-related genes and results in the initiation of conidiation under liquid culture condition.

10.1128/mBio.03007-21.2 FIG S2 Gene Ontology (GO) term enrichment analysis of differentially expressed genes (fold changes ≥ 2.0) in the RNA-seq database. Download FIG S2, TIF file, 1.6 MB. Copyright © 2021 Ren et al. 2021 Ren et al. https://creativecommons.org/licenses/by/4.0/ This content is distributed under the terms of the Creative Commons Attribution 4.0 International license.

10.1128/mBio.03007-21.3 FIG S3 (A and C) Comparison of RNA-seq data in heat map for the previously reported conidiation negative regulator-encoding genes and the selected Ca^2+^ transporter-related genes between the Δ*hapB* and WT strains cultured for 48 h in liquid MM. (B and D) The relative transcript levels of the indicated genes were further confirmed by RT-qPCR. Statistical significance was determined by Student’s *t* test. *, *P < *0.05; **, *P < *0.01; ns, not significant. Download FIG S3, TIF file, 2.1 MB. Copyright © 2021 Ren et al. 2021 Ren et al. https://creativecommons.org/licenses/by/4.0/ This content is distributed under the terms of the Creative Commons Attribution 4.0 International license.

10.1128/mBio.03007-21.9 DATA SET S1 The RNA-seq data sets of WT and Δ*hapB* strains. Download Data Set S1, XLS file, 4.5 MB. Copyright © 2021 Ren et al. 2021 Ren et al. https://creativecommons.org/licenses/by/4.0/ This content is distributed under the terms of the Creative Commons Attribution 4.0 International license.

### CBC binds to the promoters of genes related to asexual conidiation.

**FIG 3:**
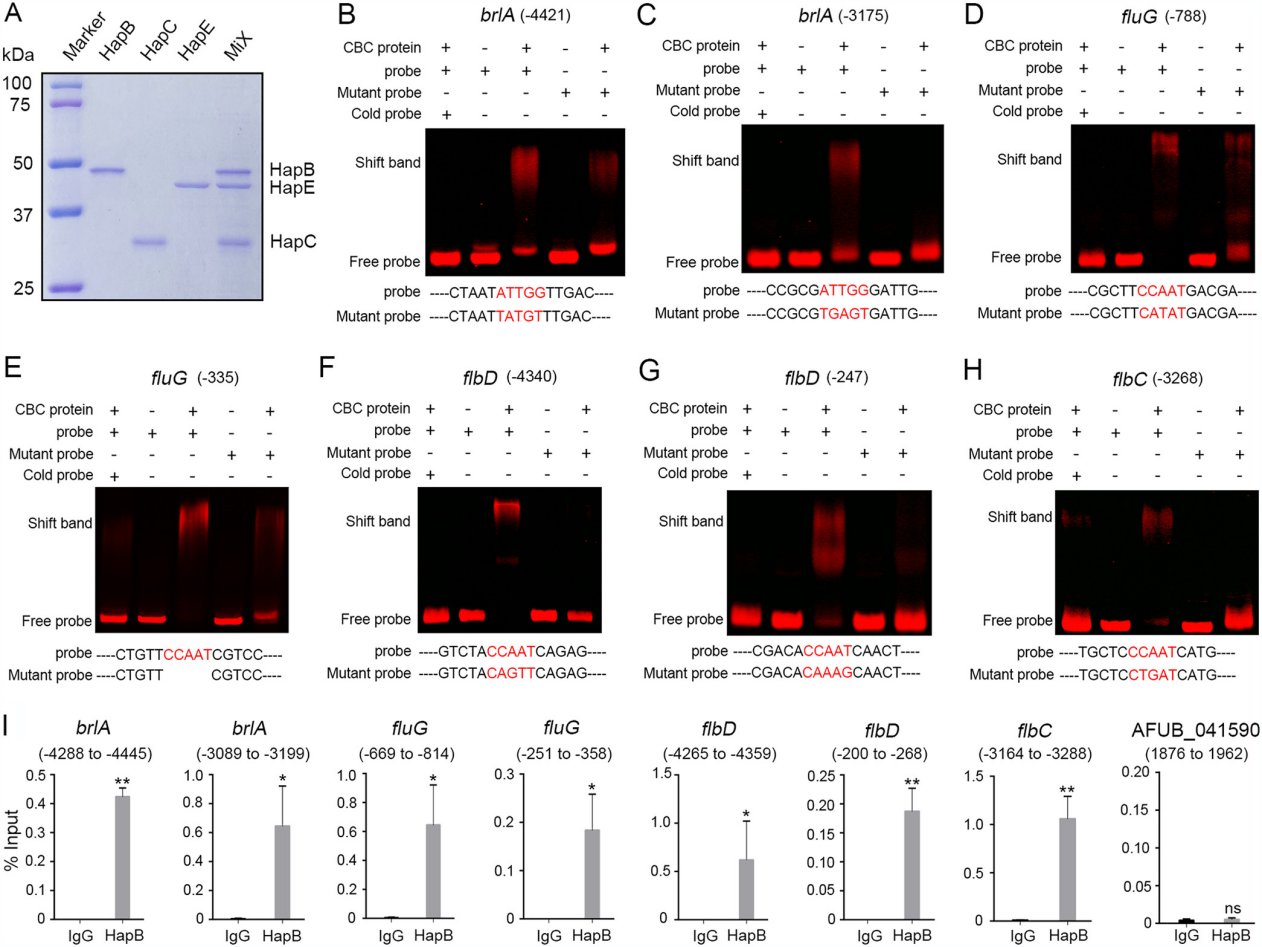
CBC *in vitro* (EMSA) and *in vivo* (ChIP-qPCR) binds to the promoters of genes related to asexual conidiation. (A) The purified HapB, HapC, and HapE proteins are shown by SDS-PAGE and Coomassie blue staining. HapB, HapC, HapE, and their mixture (1:1:1) are shown as labeled, respectively. (B to H) CBC binds to the promoters of target genes *in vitro*. EMSA of CBC binding to Cy5-labeled promoter fragments of *brlA*, *fluG*, *flbD*, and *flbC*. Two specific *brlA* probes (double-stranded DNA) were designed to target the CCAAT motif located at position bp 4421/3175 upstream of the *brlA* translational start site (−4421/−3175). Similarly, probes for *fluG*, *flbD*, and *flbC* targeting the CCAAT motif located at their own promoters were given. The specificity of EMSA binding was validated by adding specific competitors/cold probes (unlabeled probes) or substituting the Cy5-labeled mutant probe for the original Cy5-labeled probe with the CCAAT motif. (I) *In vivo* binding of the CBC was confirmed by comparing % recovery of DNA (ChIP-qPCR) from promoter fragments of conidiation-related genes to an unbound region of the AFUB_041590. ChIP-qPCR with IgG was performed as the control. Mean ± SD (*n* = 3). Unpaired *t* test. **, *P < *0.01; *, *P < *0.05; ns, not significant.

Next, we investigated how CBC affects expression of the conidiation-related genes to regulate conidiation. Since previous studies have shown that CBC can regulate the expression of targets by binding to 5′-CCAAT-3′ within the promoter regions of these target genes (35, 41, 42), we then searched the promoter sequences of the abovementioned genes that are upregulated in the absence of CBC subunit HapB. Interestingly, a motif search analysis revealed that the majority of conidiation-regulatory genes, including *brlA*, have CCAAT motifs in the promoter region. To further validate whether the A. fumigatus CBC *in vitro* could bind to the CCAAT motifs within the promoter regions of these genes, electrophoretic mobility shift assays (EMSAs) were carried out in which C-terminally His-tagged HapB, HapC, and HapE proteins were expressed in Escherichia coli (Fig. 3A) and then purified by affinity chromatography. Double-stranded oligomer probes harboring the CCAAT motif were amplified from the promoter regions of selected differentially expressed genes (*fluG*, *flbC*, *flbD*, and *brlA*) by PCR and labeled with Cy5. The results showed that probes (−4421 and −3175 of *brlA*, −788 and −335 of *fluG*, −4340 and −247 of *flbD*, and −3268 of *flbC*) mixed with the protein mixture (CBC) composed of HapB, HapC, and HapE (1:1:1, molar ratio) markedly displayed slowed-shift bands compared to the free probe without CBC, suggesting the *in vitro* binding of CBC to the CCAAT motif-containing promoter fragments of *brlA*, *fluG*, *flbD*, and *flbC* (Fig. 3B to H). Excess unlabeled probe (cold probe) significantly reduced the binding activity of CBC with the Cy5-labeled probe. The mutant probe without the CCAAT motif showed a clear decrease in binding activity to the CBC, indicating that the interaction between the protein and the DNA specifically occurs via the CCAAT motif. In comparison, CBC was unable to bind to probes at the site of −1587 of *brlA* and −1027 of *flbC* (Fig. S6A), suggesting not all probes with CCAAT motifs could directly bind to CBC *in vitro*. Next, we performed chromatin immunoprecipitation-quantitative PCR (ChIP-qPCR) experiments to verify *in vivo* binding of HapB with DNA fragments by using a green fluorescent protein (GFP)-HapB-expressing strain. The GFP-Trap agarose beads were used to perform ChIP, and then the HapB-bound DNA fragments were amplified by primers flanking the specific CCAAT regions. As a result, ChIP-qPCR data suggest that GFP-HapB was able to pull down the target templates that could be amplified to generate positive PCR products by using primers from the specific promoter regions (Fig. 3I), which cover the previously EMSA-identified CCAAT motifs. These results collectively suggest that the CBC member HapB is capable of binding to promoters of *brlA*, *fluG*, *flbD*, and *flbC*.

10.1128/mBio.03007-21.6 FIG S6 (A and B) EMSA for CBC binding to Cy5-labeled promoter fragments of *brlA*, *flbC*, and *crzA*. Probes were designed to target the CCAAT motif located at bp position 1587/1027/1109 (−1587/−1027/−1109) upstream of the *brlA/flbC/crzA* translational start site. The binding of CBC to the CCAAT motif at −3175/−4340 of *brlA*/*flbD* showed the positive binding. (C) A total of 3.5 × 10^6^ spores was grown in liquid MM at 37°C for 96 h. The relative melanin content of mycelial pellets was given. (D) The number of conidia produced by the indicated strains grown in liquid medium. The indicated strains were grown in liquid MM with or without 10 mM CaCl_2_ for 96 h in a shaker. The culture was filtered by sterile lens-cleaning paper. Then, 500 μl of the filtered supernatant was inoculated onto solid MM. The number of resulting CFU indicates the conidial amount in the inoculated culture. Statistical significance was determined by Student’s *t* test. *, *P < *0.05; **, *P < *0.01. Download FIG S6, TIF file, 0.3 MB. Copyright © 2021 Ren et al. 2021 Ren et al. https://creativecommons.org/licenses/by/4.0/ This content is distributed under the terms of the Creative Commons Attribution 4.0 International license.

### *brlA* and *flbC* are required for the submerged conidiation induced by a lack of *hapB*.

**FIG 4:**
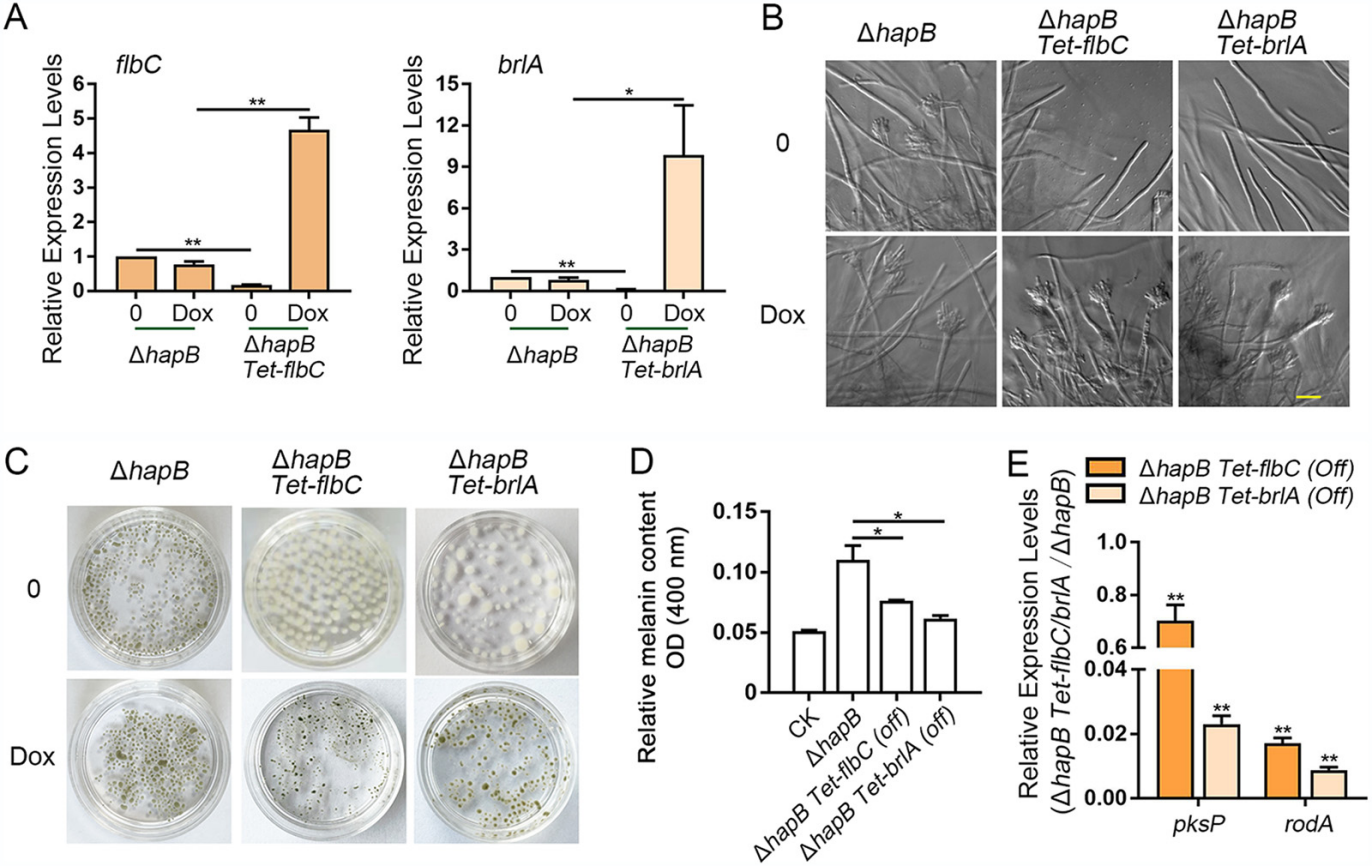
The conidiation-related genes *brlA* and *flbC* were required for asexual reproduction in the *hapB* mutant in submerged culture. (A and E) The transcript levels of the indicated genes in the Δ*hapB*, Δ*hapB Tet-flbC*, and Δ*hapB Tet-brlA* strains grown in liquid MM with or without 0.6 μM doxycycline (Dox) at 37°C for 48 h in a shaker culture. (B to D) A total of 3.5 × 10^6^ spores were grown in liquid MM with or without 0.6 μM Dox at 37°C for 96 h. Asexual conidiogenous structure, macromorphology, and melanin of mycelial pellets were observed and measured. “(Off)” indicates the condition without Dox treatment. Scale bar represents 10 μm. Statistical significance was determined by Student’s *t* test. *, *P < *0.05; **, *P < *0.01; ns, not significant.

To further explore whether the submerged conidiation of the Δ*hapB* mutant was related to the expression of central and upstream conidiation genes, *brlA* and *flbC* were conditionally expressed under the control of the Tet on-off system in the Δ*hapB* mutant, yielding Δ*hapB Tet-flbC* and Δ*hapB Tet-brlA*, respectively. In this system, the gene was turned on in the presence of doxycycline and was turned off in the absence of doxycycline. The results of the real-time quantitative PCR (RT-qPCR) analysis showed that under repression conditions the mRNA expression of *flbC* and *brlA* was significantly reduced to 17% and 10%, respectively, in the Δ*hapB Tet-flbC* and Δ*hapB Tet-brlA* mutants, compared to the Δ*hapB* mutant (Fig. 4A), indicating that *flbC* and *brlA* could be significantly decreased by this Tet-off system. Next, we tested the conidiation of related strains grown in liquid medium and found that the submerged conidiation caused by loss of *hapB* was completely abolished in conditionally repressed *flbC* or *brlA* strains (Fig. 4B). Accordingly, repression of *flbC* or *brlA* markedly reduced the relative melanin accumulation accompanied by low expression of the melanin synthesis-related gene *pksP* and hydrophobin-encoding gene *rodA* (Fig. 4C to E), especially in the Δ*hapB Tet-brlA* strain, which is consistent with the abovementioned conidiation phenotype. In comparison, the conditional strains under the doxycycline-induced condition showed significant pigmentation accumulation in mycelium pellets and the visible asexual conidiogenous structure (Fig. 4B and C). These results suggest that the submerged conidiation caused by *hapB* deficiency is dependent on the *flbC-* and *brlA-*associated regulatory pathways.

### External calcium enhances conidiation caused by the absence of CBC.

**FIG 5:**
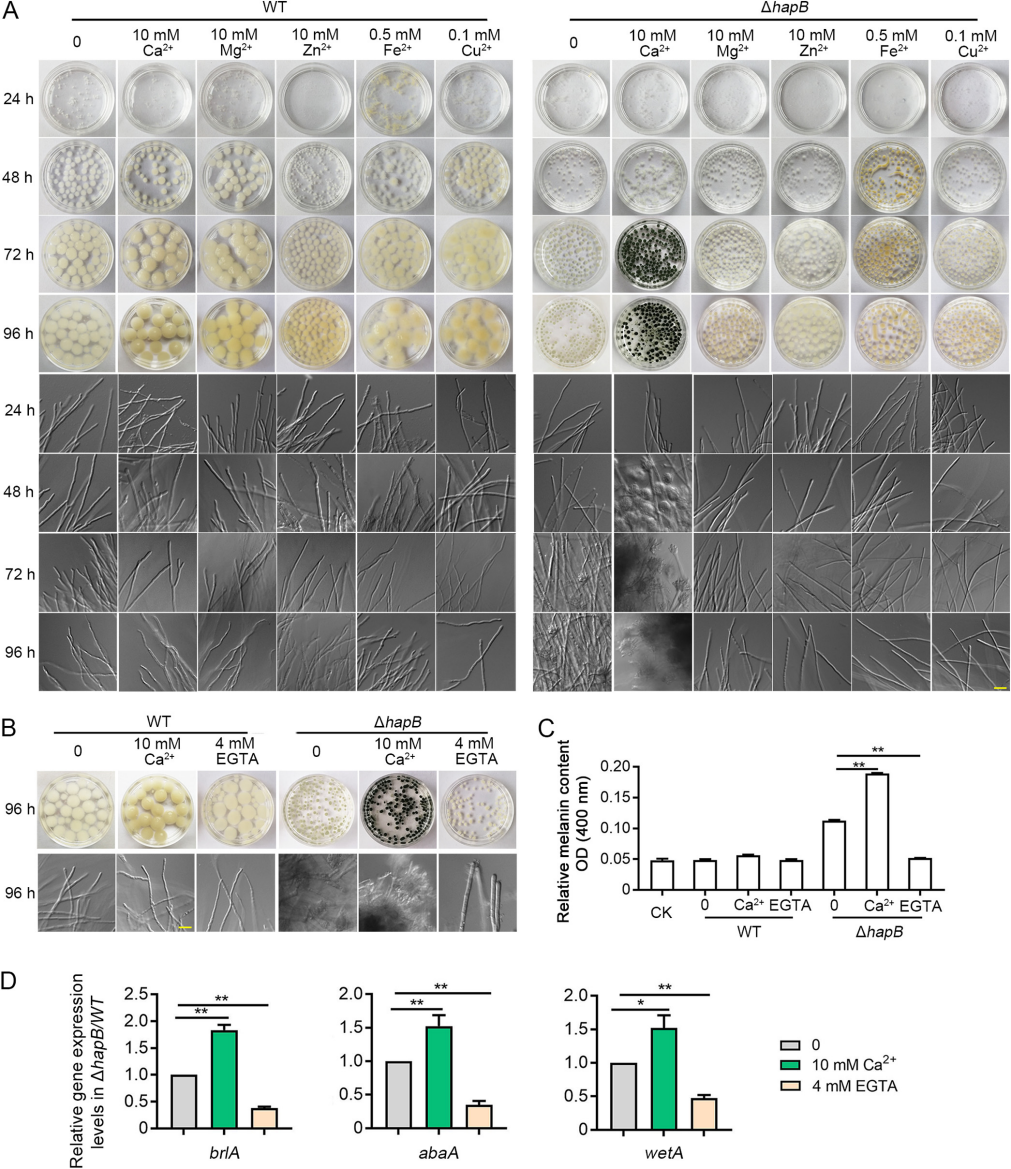
Calcium enhanced the submerged conidiation of the CBC mutants. (A to C) A total of 3.5 × 10^6^ spores were grown in liquid MM supplemented with indicated reagents in a shaker at 37°C for the indicated times, and the macromorphology, asexual conidiogenous structure, and melanin content of mycelial pellets were observed and measured. Scale bar represents 10 μm. (D) The relative transcript levels of the indicated genes between the Δ*hapB* and WT strains grown in liquid MM with/without 10 mM CaCl_2_ or 4 mM EGTA at 37°C for 48 h. Statistical significance was determined by Student’s *t* test. *, *P < *0.05; **, *P < *0.01.

To further dissect the molecular mechanism by which CBC negatively regulates conidiation under liquid culture conditions, label-free quantitative proteomics analysis was performed to investigate differentially abundant proteins between WT and Δ*hapB* strains grown in liquid minimal medium. The results showed that a total of 3,892 proteins were detected and quantified, and proteins with 2-fold change ratios between Δ*hapB* and WT strains (2.0 ≤ Δ*hapB/*WT strain ≤ 0.50) were selected for GO annotation and enrichment analysis. The expression levels of 429 and 355 proteins were increased and decreased, respectively, in the Δ*hapB* mutant compared to the WT strain (Data Set S2). GO enrichment analysis according to molecular function (MF) demonstrated that a large number of ion binding proteins, especially metal ion binding proteins, were enriched in the differentially expressed proteins between the WT and Δ*hapB* strains (Fig. S4). To further verify whether metal ions could affect submerged conidiation in the CBC mutants, the WT and Δ*hapB* strains were grown in liquid minimal medium supplemented with different divalent metal ions, including Ca^2+^, Mg^2+^, Zn^2+^, Fe^2+^, and Cu^2+^. As shown in Fig. 5A, when the Δ*hapB* strain was exposed to additional calcium (10 mM) in minimal medium, it displayed much more deep green conidia and conidiophores in mycelial pellets than when grown in MM with other noncalcium divalent metal ions, suggesting that calcium was able to enhance abnormal asexual conidiation caused by a lack of *hapB*. In contrast, when the 4 mM Ca^2+^-chelating agent EGTA was added to the medium, the submerged conidiation of the CBC mutants in liquid culture was almost abolished and the mycelial pellets were pale (Fig. 5B and C and Fig. S5A). As shown in Fig. 5D, the mRNA expression of *brlA*, *abaA*, and *wetA* in the Δ*hapB* strain was significantly upregulated by the addition of Ca^2+^ and downregulated by supplementation with EGTA. These data collectively indicate that calcium enhances the submerged conidiation of the CBC mutants. Notably, there was no detectable difference for the colony phenotype in CBC mutants on solid medium between the treatment of CaCl_2_ and EGTA under the tested concentration (Fig. S5B to D).

10.1128/mBio.03007-21.4 FIG S4 Gene Ontology (GO) term enrichment (top 20) analysis of differentially expressed proteins (fold changes ≥ 2.0) in the proteomics database. Based on molecular function (MF), the top 20 GO terms were identified. Download FIG S4, TIF file, 0.9 MB. Copyright © 2021 Ren et al. 2021 Ren et al. https://creativecommons.org/licenses/by/4.0/ This content is distributed under the terms of the Creative Commons Attribution 4.0 International license.

10.1128/mBio.03007-21.5 FIG S5 (A) A total of 3.5 × 10^6^ spores was grown in liquid MM with or without 10 mM CaCl_2_ or 4 mM EGTA in a shaker at 37°C for 4 days, and the macromorphology and asexual conidiogenous structure of mycelial pellets were observed. Scale bar represents 10 μm. (B to D) Spores (2 × 10^5^) were inoculated on solid MM with or without 10 mM CaCl_2_ or 4 mM EGTA at 37°C for 3 days. Morphology, diameter, and conidial amount of the resulting colonies. Statistical significance was determined by Student’s *t* test. ns, not significant. Download FIG S5, TIF file, 1.8 MB. Copyright © 2021 Ren et al. 2021 Ren et al. https://creativecommons.org/licenses/by/4.0/ This content is distributed under the terms of the Creative Commons Attribution 4.0 International license.

10.1128/mBio.03007-21.10 DATA SET S2 The proteomics data sets of WT and Δ*hapB* strains. Download Data Set S2, XLSX file, 0.3 MB. Copyright © 2021 Ren et al. 2021 Ren et al. https://creativecommons.org/licenses/by/4.0/ This content is distributed under the terms of the Creative Commons Attribution 4.0 International license.

### Lack of HapB causes enhanced transient cytosolic Ca^2+^ levels and activates the Ca^2+^-calcineurin-CrzA pathway to enhance submerged conidiation.

**FIG 6:**
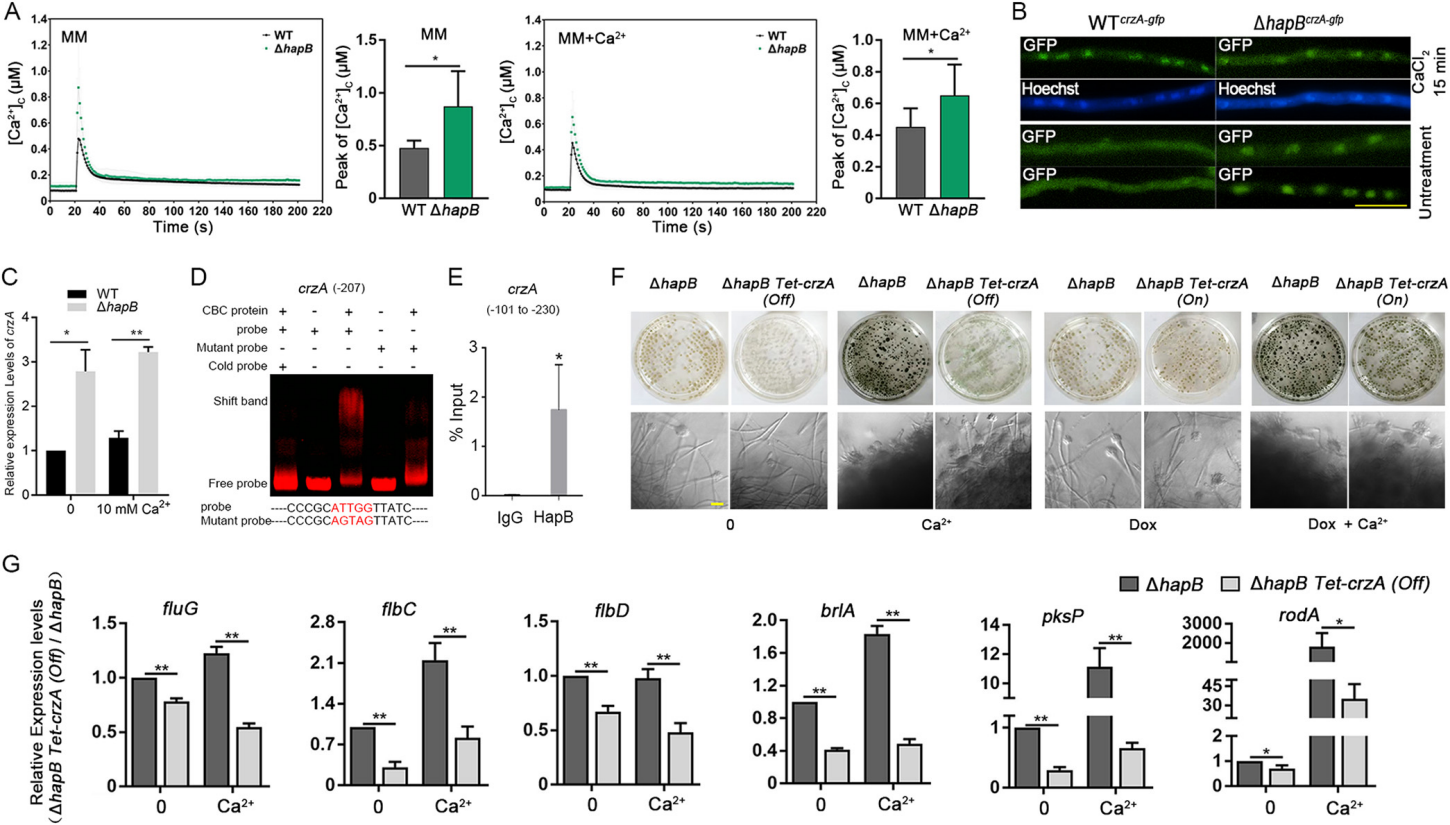
Lack of HapB led to enhanced transient cytosolic Ca^2+^ levels and activated the Ca^2+^-calcineurin-CrzA pathway. (A) The aequorin-expressing strains were stimulated with 100 mM CaCl_2_ after growth in MM with or without 2 mM CaCl_2_. The linear graphs indicated that the real-time [Ca^2+^]_c_ changes in response to calcium stimuli. [Ca^2+^]_c_, free Ca^2+^ concentration in the cytoplasm. Basal [Ca^2+^]_c_ was indicated by the resting level prior to extracellular calcium stimulus at the 20-s time point. [Ca^2+^]_c_ amplitude was indicated by the poststimulatory peak value of [Ca^2+^]_c_. Data are the average from at least six experiments. (B) Epifluorescence microscopic images demonstrating the CrzA-GFP distribution under untreated conditions (lower panels) or treatment with CaCl_2_ (100 mM) for 15 min (upper panels) in the parental wild-type and Δ*hapB* strains. Hoechst is a nuclear localization signal dye used to visualize the nucleus. Scale bar represents 10 μm. (C and G) The relative transcript levels of the indicated genes in indicated strains grown in liquid MM with/without 10 mM CaCl_2_ for 48 h. (D) EMSA of CBC binding to the CCAAT motif at the −207 position of *crzA* promoter. (E) ChIP-qPCR analysis for binding affinity of CBC to the specific promoter fragments of *crzA* in the GFP-HapB-expressing strain. ChIP-qPCR with IgG was performed as for the control. (F) A total of 3.5 × 10^6^ spores were grown in liquid MM for 36 h, and then the resulting mycelial pellets were transferred into new liquid MM with or without 10 mM CaCl_2_, and/or 10 μg/ml Dox was added to the medium for 60 h. Asexual conidiogenous structure and macromorphology of mycelial pellets were observed. Scale bar represents 10 μm. Statistical significance was determined by Student’s *t* test. *, *P < *0.05; **, *P < *0.01.

We next investigated whether CBC affects calcium homeostasis in A. fumigatus. As shown in Fig. S3C and D, using RT-qPCR, we measured the mRNA expression of the known Ca^2+^ transporter-encoding genes *midA*, *cchA*, *figA*, *vcxA* to -*E*, *pmcA* to -*C*, *pmrA*, *srcA*, *mcuA*, and *yvcA* and found that the majority of these genes in the Δ*hapB* mutant truly exhibited upregulation to a certain extent compared with those in the WT strain, which is consistent with the RNA-seq data. Of these upregulated genes, the majority of Ca^2+^ transporters were involved in calcium influx into the cytosol or calcium stores in vacuoles and mitochondria. Therefore, HapB deficiency could affect cellular calcium homeostasis. To further test this hypothesis, we constructed aequorin-containing related strains for real-time monitoring of the dynamics of free cytosolic Ca^2+^ ([Ca^2+^]_c_) in living hyphal cells. As shown in Fig. 6A, the [Ca^2+^]_c_ amplitude of the *hapB* mutant strain was significantly increased compared with that of the WT strain, suggesting that the absence of HapB resulted in abnormally higher cellular calcium capacity than that in the parental wild type. It has been reported that cytosolic free Ca^2+^ can bind to calmodulin, a predominant calcium sensor protein that activates protein phosphatase 2B (calcineurin), which subsequently dephosphorylates and activates the transcription factor CrzA, inducing its nuclear translocation (43–46). We speculated that HapB may have an effect on CrzA translocation and then generated C-terminally GFP-labeled CrzA (CrzA-GFP) strains on the wild-type and *hapB* mutant backgrounds. As shown in Fig. 6B, CrzA-GFP was located in the cytosol in minimal medium, as expected when CaCl_2_ was added to medium, and almost all of the CrzA-GFP was translocated into nuclei in the WT strain. However, in the *hapB* mutant strain, the CrzA-GFP strain showed a constant nuclear localization pattern in either calcium-supplemented medium or minimal medium alone, suggesting that *hapB* deficiency may lead to constitutive nuclear localization of the calcium-responsive transcription factor CrzA. In addition, we found that deletion of *hapB* also increased expression of *crzA* to some extents at the transcriptional level (Fig. 6C). To further test whether HapB could bind to the promoter of CrzA, through a CCAAT motif search analysis, we revealed that the putative *crzA* promoter has two CCAAT motifs located at position 1,109/207 bp (−1109/−207) upstream of the *crzA* translational start site. EMSA showed that CBC could *in vitro* bind to the CCAAT motif at the position −207, but not at the position −1109 (Fig. 6D and Fig. S6B). Furthermore, ChIP-qPCR verified that HapB *in vivo* bound to the specific region (position: −101 to −230) in the *crzA* promoter (Fig. 6E). Collectively, HapB not only affects localization of CrzA in A. fumigatus cells but also regulates *crzA* expression via binding to its promoter.

We next speculated whether the constant nuclear localization of CrzA contributed to submerged conidiation in the *hapB* mutant strain under liquid culture condition. To test this hypothesis, we generated a conditionally expressed CrzA strain (Δ*hapB Tet-crzA*) under the control of the Tet on–off system on the background of *hapB* deletion. As shown in Fig. 6F and Fig. S6C and D, repressing *crzA* expression significantly reduced, but did not completely abolish, the submerged conidiation and pigmentation induced by the absence of *hapB*, in particular when calcium was present. By comparison, the Δ*hapB* Tet-*crzA* strain under the doxycycline-induced condition showed similar phenotypes as the Δ*hapB* strain (Fig. 6F), suggesting that Tet-on state of *crzA* is functional. Furthermore, turning off *crzA* in the Δ*hapB* strain (Δ*hapB Tet-crzA*) led to decreased expression of conidiation-, pigment-, and hydrophobin-related genes, including *fluG*, *flbC*, *flbD*, *brlA*, *pksP*, and *rodA* (Fig. 6G), which corresponds to the phenotypic difference. These data suggest that calcium-enhanced submerged conidiation of the *hapB* mutant is primarily dependent on CrzA.

## DISCUSSION

The life cycle of filamentous fungi generally consists of hyphal growth and developmental process to generate asexual spores (1, 16, 47). For fungal pathogens, hyphal growth is required for host invasion. Under suboptimal growth conditions, fungi will undergo genetic reprogramming and initiate asexual conidiation, allowing dissemination to new hosts and the beginning of a new infection cycle. Thus, both vegetative hyphal growth and asexual reproduction are critical for invasion, growth, spore dissemination, and virulence in hosts for mycosis (17, 25). In comparison, for industrially important fungi, optimized liquid fermentation processes for biomass production are required (48–51). During this process, the initiation of asexual reproduction is repressed through the suppression of the developmental inductive transcription factor gene *brlA*, a conserved essential conidiation gene in fungi, and its upstream developmental activation pathway (27). Previous studies have verified that in the model filamentous fungus A. nidulans or A. fumigatus, dysfunction of NsdC, NsdD, MpkB, VosA, SfgA, VeA, or VelB results in the activation of conidiation after a period of vegetative growth under liquid culture conditions (26–28, 30, 34), suggesting that negative regulators exist for suppressing submerged conidiation to maintain constant vegetative growth with increasing mycelial pellets.

In this study, we report a novel negative regulator, the CCAAT-binding complex (CBC), of conidiation in liquid-submerged culture in A. fumigatus. CBC is a heterotrimeric transcription factor comprising three subunits (HapB/HapC/HapE) and is highly conserved from yeast to humans (41, 52–54). In A. fumigatus, we verified that CBC is able to bind to the CCAAT motif within the promoter of *brlA*, a key regulator of asexual development (conidiation), and its upstream elements *fluG*, *flbC*, and *flbD* to suppress the expression of these genes (8). Lack of CBC subunit HapB causes desuppression of conidiation in conjunction with the action of the calcium transcription factor CrzA required for *brlA* overexpression in liquid-submerged culture. Furthermore, we found that deletion of *hapB* resulted in abnormal cellular calcium capacity and that calcium remarkably enhanced the submerged conidiation caused by CBC dysfunction. Notably, *hapB* deletion leads to constant nuclear localization of CrzA-GFP, and CrzA is required for calcium-enhanced conidiation in the *hapB* mutant, implying that deletion of the CBC subunit results in calcium-enhanced constitutive activation of CrzA. These data collectively indicate that the transcription factors CrzA and CBC are reverse regulators of key conidiation regulator *brlA* in response to developmental signals. The findings of this study have explored the molecular switch mechanism between vegetative growth and asexual reproduction in liquid culture. Notably, A. fumigatus CBC mutants showed similar defects as each other in radial growth and conidiation compared to the wild-type strain on solid minimal medium, which is consistent with the previous report on A. nidulans (52).

As a transcription factor, CBC binds to the 5′-CCAAT-3′ motif within the promoters of its target genes to regulate their expression. The *cis*-acting sequence CCAAT is present in approximately 30% of eukaryotic promoters (55, 56). RNA-seq data analysis revealed that almost half (46.3%) of the A. fumigatus transcripts were differentially expressed at least 2-fold between the wild-type and *hapB* mutant strains, suggesting that CBC is a potential global transcriptional regulator. Notably, a lack of HapB led to the upregulation of most of the key conidiation regulatory genes, including *brlA*, which encodes a master regulator of the conidiation pathway. BrlA is a key regulator of conidiation in A. nidulans, A. fumigatus, and other filamentous fungi, and the appropriate expression of conserved *brlA* is necessary for the transition from vegetative growth to asexual development. In liquid-submerged culture, *brlA* is expressed at a low level, while a lack of CBC subunit HapB causes abnormally high *brlA* expression and results in submerged conidiation. Our EMSA data demonstrated that CBC was able to directly bind the CCAAT motif within the promoters of the conidiation inducer genes *brlA*, *fluG*, *flbC*, and *flbD*. Accordingly, a previous study revealed that CBC-associated ChIP-seq data sets showed that *brlA*, *fluG*, *flbD*, and *flbC* have at least one HapC-binding peak upstream of their translational start site (53).

Comparison data analyzed in EMSA of this study indicate that some sites/CCAAT motifs (−4421 and −3175 of *brlA*, −4340 of *flbD*, and −3268 of *flbC*) are in close proximity to the *in vivo* HapC-binding peaks (−4445 and −3192 of *brlA*, −4327 of *flbD*, and −3230 of *flbC*) reported in previous CBC-associated ChIP-seq data sets (53), but others (−1578 of *brlA*, −788 and −335 of *fluG*, −247 of *flbD*, and −1027 of *flbC*) are relatively far from the nearest HapC-binding peaks (−2603 of *brlA*, −634 and −592 of *fluG*, −725 of *flbD*, and −1548 of *flbC*). Results displayed that, except for −1578 of *brlA* and −1027 of *flbC*, all the other CCAAT motifs can *in vitro* bind to CBC. These data revealed that the *in vitro* binding sites of CBC in EMSA are not exactly but mostly matched to sites previously reported for the HapC-binding peak *in vivo*. Furthermore, through ChIP-qPCR, we tested the *in vivo* binding of CBC to the CCAAT motifs in relative genes and verified the binding of HapB to the specific promoter regions that harbor the abovementioned CBC-binding CCAAT motifs in EMSA. Therefore, the data collectively demonstrate that CBC is consistently able to *in vivo* and *in vitro* bind to CCAAT motifs of indicated genes.

These results indicated that CBC directly regulates both upstream and central conidiation regulators. In line with this evidence, repressing *brlA* or *flbC* expression completely abolished the submerged conidiation caused by the *hapB* deletion (Fig. 4B and C). These results collectively indicated that the abnormal upregulation of *brlA* and *flbC*, involved in the upstream and central pathway of conidiation, contributes to submerged conidiation in the CBC mutants. However, based on these data alone, we could not exclude other possible mechanisms whereby submerged conidiation induced by the CBC mutants is due to derepression of other negative regulators of conidiation. In A. nidulans or A. fumigatus, it has been reported that the *vosA*, *veA*, *velB*, *sfgA*, *nsdC*, *nsdD*, and *mpkB* mutants also displayed asexual conidiation phenotypes similar to those of the CBC mutants in liquid medium (26–28, 30, 34). We then compared the expression differences of the abovementioned genes in the *hapB* mutant and its parental wild-type strain. There was no significant suppression of their expression in the *hapB* mutant compared to that in the parental wild-type strain, suggesting that the derepression of *brlA* and the submerged conidiation in the *hapB* mutant were unlikely to be attributed to these reported negative regulators of conidiation. Instead, CBC is able to directly and negatively regulate the expression of conidiation-related genes *brlA* and *flbC.*

**FIG 7:**
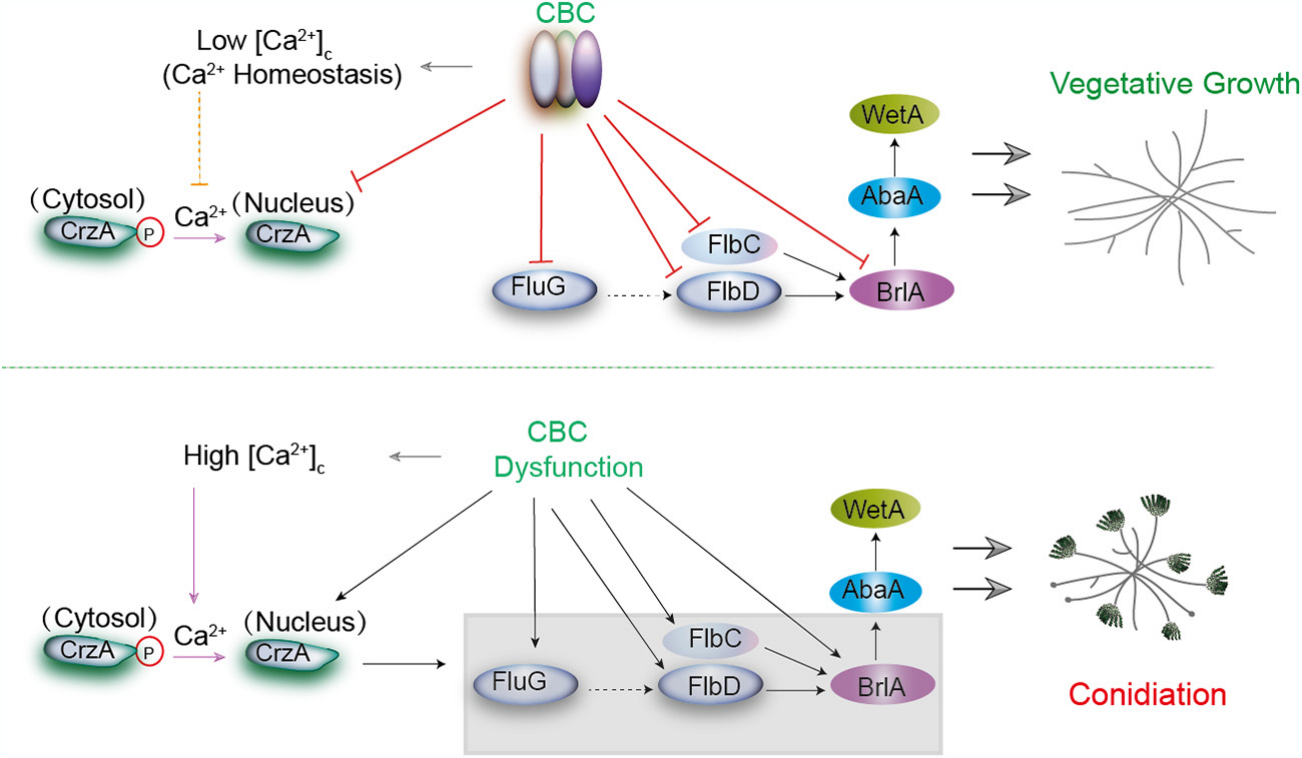
A working model showing how CBC and CrzA reversely regulate vegetative growth and asexual reproduction in liquid-submerged culture. When the transcription factor CBC is present (top), it represses the *brlA*-associated central regulatory pathway in combination with its upstream regulators *fluG*, *flbD*, and *flbC*, and *crzA* (encoding the calcium-responsive transcription factor CrzA). Meanwhile, the cytosolic Ca^2+^ concentration is at a low level (Ca^2+^ homeostasis), which represses the dephosphorylation of CrzA and its translocation to the nucleus. In this situation, A. fumigatus undergoes constant vegetative growth in liquid-submerged culture. When CBC is absent, the expressive suppression of the upstream and central regulator in the conidiation pathways and *crzA* is stopped. Additionally, the absence of CBC also causes enhanced transient cytosolic Ca^2+^ levels and activates conidiation-positive inducer Ca^2+^-CrzA modules to further elevate the expression of the indicated genes. As a result, asexual conidiation is activated.

As an intracellular second messenger in eukaryotic cells, the calcium signaling pathway plays important roles in various physiological processes, including asexual conidiation (8, 45, 57). In the present study, the submerged conidiation of CBC mutants was significantly enhanced under calcium-treated conditions and was relatively inhibited by the calcium chelator EGTA, indicating that the calcium signaling pathway is tightly associated with this submerged conidiation. A conserved Ca^2+^-calcineurin-CrzA pathway in eukaryotic cells has been widely reported to regulate growth and development. Calcium-activated calcineurin can dephosphorylate the transcription factor CrzA, which induces its translocation to the nucleus and then regulates expression of targeted genes (43–46). We found that the *hapB* deletion caused the enhanced transient cytosolic Ca^2+^ level which results in the persistent nuclear localization of CrzA. The enhanced transient cytosolic Ca^2+^ level indicated that lack of CBC would increase cellular calcium. In fact, the RNA-seq data combined with the RT-qPCR results indicated that the majority of calcium influx channels in the plasma membrane and endomembrane system had upregulated expression to some extent in the *hapB* mutant. It has been reported that enhancement of transient cytosolic Ca^2+^ levels and persistent nuclear localization of CrzA may affect its transcription activity (43, 45, 58, 59). Thus, these data suggest the *hapB* deletion may abnormally activate the transcription activity of CrzA. Notably, previously reported results along with ours demonstrated that deleting either *cnaA* (calcineurin subunit-encoding gene) or *crzA* leads to reduced *brlA* expression and conidiation in A. fumigatus. Consistently, when the expression of *crzA* was conditionally turned off, both the upstream and central conidiation regulators *fluG*, *flbC*, *flbD*, and *brlA* showed significantly low expression in the *hapB* mutant, especially under calcium-supplemented culture conditions accompanied by decreased submerged conidiation, demonstrating that Ca^2+^-calcineurin-CrzA affects conidiation by increasing the expression of conidiation-related regulators. A working model summarizing the findings of this study is shown in Fig. 7. Therefore, the present results demonstrate the molecular mechanism underlying the regulation of asexual reproduction involving a new negative regulator, CBC, in A. fumigatus.

## MATERIALS AND METHODS

### Strains and culture.

The genotype of each A. fumigatus strain is provided in the supplemental material (Table S1). For conidiation determination, RNA-seq, proteomics, and RT-qPCR, A. fumigatus strains were grown in 100 ml liquid minimal medium (MM) with or without 10 mM CaCl_2_ at 37°C for the indicated time with shaking at 220 rpm. MM comprises 1% glucose, 1 ml liter^−1^ trace elements, and 50 ml liter^−1^ 20× salt (60), pH 6.0.

10.1128/mBio.03007-21.7 TABLE S1 The list of A. fumigatus strains used in this study. Download Table S1, DOCX file, 0.02 MB. Copyright © 2021 Ren et al. 2021 Ren et al. https://creativecommons.org/licenses/by/4.0/ This content is distributed under the terms of the Creative Commons Attribution 4.0 International license.

### Plasmid construction.

The plasmid for the *hapB* complementation strain was generated as follows. The PCR experiment was performed by using the primers NotI-hapB-F and NotI-hapB-R to amplify the *hapB* gene from the genomic DNA (gDNA) of A. fumigatus. Then, the *hapB* gene was subcloned into the NotI site of p-zero-pyr4, generating plasmid p-zero-pyr4-hapB. The resulting plasmid was transformed into the Δ*hapB* deletion strain, yielding the *hapB*-complemented strain (*hapB^C^*).

### Construction of Tet-conditional and GFP-tagged strains.

The MMEJ-CRISPR system was used as described in our previously published papers to induce the Tet-On/Off system/cassette in front of the transcriptional start site of conidiation-related genes and to tag *hapB* and *crzA* with GFP at the 5′ and 3′ termini, respectively (60, 61). Briefly, single guide RNA (sgRNA) was synthesized *in vitro* using the MEGAscript T7 kit. The corresponding repair template was amplified by PCR from plasmid pCH008 (62). Then, the repair template fragments and corresponding sgRNA were cotransformed into ZC03/WT (a Cas9-expressing A. fumigatus recipient strain). The primers and annotations for the repair templates and sgRNA are listed in Table S2. Transformation procedures were performed as previously described (60).

10.1128/mBio.03007-21.8 TABLE S2 Primers used in this study. Download Table S2, DOCX file, 0.03 MB. Copyright © 2021 Ren et al. 2021 Ren et al. https://creativecommons.org/licenses/by/4.0/ This content is distributed under the terms of the Creative Commons Attribution 4.0 International license.

### RNA-seq analysis and RT-qPCR.

Fresh A. fumigatus conidia were grown in liquid MM in a rotary shaker at 220 rpm at 37°C for 48 h. For RNA-sequencing (RNA-seq) analysis, mycelial pellets were collected and quickly frozen in liquid nitrogen. After mRNA extraction, purification, and library construction, sequencing was performed by next-generation sequencing (NGS) based on the Illumina sequencing platform. A fold change of ≥2 and a *P* value of < 0.05 were set as the threshold values for differentially expressed genes. The detailed procedures were performed by Shanghai OE Biotech. Co., Ltd. (China). Each sample was analyzed using three biological repetitions. For RT-qPCR analysis, total RNA was extracted with the UNlQ-10 Column TRIzol total RNA isolation kit (Sangon Biotech, B511361) according to the manufacturer’s directions. The HiScript II Q RT SuperMix for qPCR kit (Vazyme, R223-01) was used to synthesize cDNA. Independent assays were performed with three replicates, and transcript levels were calculated by the comparative threshold cycle (Δ*C_T_*) and normalized against the mRNA expression of *tubA* in A. fumigatus. The 2^−ΔΔ^*^CT^* method was used to determine the changes in mRNA expression. All the RT-qPCR primers are given in Table S2.

### Comparative label-free quantitative proteomics analysis.

Fresh A. fumigatus conidia were grown in liquid MM in a rotary shaker at 220 rpm at 37°C for 48 h. Quantitative proteomic analysis was performed at Wuhan Genecreate Biological Engineering Co., Ltd., as a commercial service. Samples were first ground to powder in liquid nitrogen and incubated in lysis buffer (7 M urea, 2 M thiourea, 4% SDS, 40 mM Tris-HCl, pH 8.5) containing 1 mM phenylmethylsulfonyl fluoride (PMSF) and 2 mM EDTA for 5 min. Then, 10 mM dithiothreitol (DTT) was added to the sample. The suspension was sonicated for 15 min on ice and then centrifuged at 4°C and 13,000 rpm for 20 min. The supernatant was mixed with 4 volumes of precooled acetone at −20°C overnight. After centrifugation, the protein pellets were air dried and resuspended in 8 M urea-100 mM triethylammonium bicarbonate (TEAB) (pH 8.0). Protein samples were reduced with 10 mM DTT at 56°C for 30 min and alkylated with 50 mM iodoacetamide (IAM) at room temperature for 30 min in the dark. After dilution 4 times with 10 mM TEAB, the total protein concentration was measured using the Bradford method. Equal amounts of proteins from each sample were digested with trypsin. After digestion, peptides were desalted using C_18_ columns, and the desalted peptides were dried with a vacuum concentration meter. The dried peptide powder was redissolved in 20 μl with 0.5 M TEAB for peptide labeling. The dried peptide samples were reconstituted with mobile phase A (2% acetonitrile [ACN], 0.1% formic acid [FA]) and centrifuged at 20,000 × *g* for 10 min, and the supernatant was taken for injection. Separation was performed on a Thermo UltiMate 3000 ultrahigh-performance liquid chromatograph (UHPLC). The sample was first enriched in a trap column and desalted and then transferred to a tandem self-packed C_18_ column and separated. The nanoliter liquid phase separation end was directly connected to the mass spectrometer.

The peptides separated by liquid phase chromatography were ionized by a nano-electrospray ionization (nanoESI) source and then passed to a Q-Exactive HF X tandem mass spectrometer for data-dependent acquisition (DDA) mode detection. The resulting tandem mass spectrometry data were processed using the MaxQuant (1.6.10.43) search engine. Tandem mass spectra were searched against the Aspergillus UniProt database concatenated with the reverse decoy database. Proteins with change ratios significantly different from general protein variation (2.0 ≤ Δ*hapB*/wild type ≤ 0.50) were analyzed for GO terms and by KEGG pathway analysis using the UniProt-GOA database (http://www.ebi.ac.uk/GOA/) and the KEGG database, respectively.

### Measurement of the free Ca^2+^ concentration ([Ca^2+^]_c_).

The strains expressing aequorin were cultured for 2 days at 37°C to form fresh spores. Fresh spores were filtered through nylon cloth and washed 10 times in distilled deionized water. One million (10^6^) spores in 100 μl liquid MM with/without 2 mM CaCl_2_ were inoculated into each well of a 96-well microtiter plate (Thermo Fisher) and incubated at 37°C for 24 h. The subsequent measurement of the free Ca^2+^ concentration was performed as described previously (63, 64).

### Melanin measurement.

Since the melanin could be easily dissolved in alkaline solution (65, 66), we extracted the melanin from mycelial pellets using NaOH lysis buffer. After that, the spectrophotometric method based on the absorption of light at a wavelength (400 nm) for melanin is the most popular method used for the measurement of the relative melanin contents between different samples. In brief, fresh A. fumigatus conidia were grown in liquid MM in a rotary shaker at 220 rpm at 37°C for 96 h. The resulting mycelial pellets were quickly collected and frozen in liquid nitrogen. Fifty milligrams of powder was added to 1 ml of 2 M NaOH. The suspension liquid was incubated at 37°C for 2 weeks and centrifuged at 12,000 × *g* for 10 min. After diluting twice, the optical density of the supernatant at 400 nm (OD_400_) was measured by spectrophotometry. The OD_400_ values were used to compare the relative melanin contents.

### Recombinant CBC protein purification and electrophoretic mobility shift assay (EMSA).

To express His-labeled CBC subunits in Escherichia coli, the exons of *hapB*, *hapC*, and *hapE* (three subunits of CBC) were amplified with three pairs of primers, EmsA-hapB-F/EmsA-hapB-R, EmsA-hapC-F/EmsA-hapC-R, and EmsA-hapE-F/EmsA-hapE-R, respectively, and then ligated into the pET30a vector. The resulting vector was subsequently transformed into BL21(DE3) competent cells. The resulting strains were grown in LB medium at 37°C to an OD_600_ (optical density, measured at 600 nm) between 0.6 and 0.8 and subsequently induced by 0.1 mM isopropyl-β-d-thiogalactoside. Target proteins were purified as previously described (67) using a rapid Ni-nitrilotriacetic acid (NTA) agarose minicolumn. EMSA was performed as previously described with minor modifications (67, 68). The basic reaction mixtures consisted of 6 μl of 5× EMSA binding buffer, 1.5 μl of 1 mg/ml salmon sperm DNA, 60 ng Cy5-labeled probe (double-stranded DNA), 0.5 μg HapB, 0.8 μg HapC, and 0.6 μg HapE. For competitive testing, a 30-fold nonlabeled DNA probe (1,800 ng) as a competitive cold probe was added to the basic reaction mixtures. To confirm the specific binding of CBC to the CCAAT motif, the CCAAT motif within the probe was randomly mutated into a non-CCAAT sequence. The reaction mixtures were incubated at 37°C for 30 min and then separated in a 5% polyacrylamide gel in 0.5× Tris-borate=EDTA buffer. The Cy5-labeled probes were detected with an Odyssey machine.

### Fluorescence microscopy.

Fresh conidia of CrzA-GFP-expressing strains in 0.5 ml of liquid MM were grown under different treatments (see figure legends) on sterile glass at 37°C for indicated times. The resulting hyphae were gently washed with phosphate-buffered saline (PBS) buffer three times and then fixed with 4% paraformaldehyde for 1 h. To observe nuclei, the hyphae were stained with Hoechst 33528 at a final concentration of 100 μg/ml for 30 min. For microscopic observation of conidiophore formation, fresh spores were grown in liquid medium at 37°C with shaking at 220 rpm for indicated times, and then the resulting mycelial pellets sandwiched between the microscope slide and coverslips were observed directly by microscopy. The fluorescent images were captured with a Zeiss Axio Imager A1 microscope (Zeiss, Jena, Germany).

### ChIP-qPCR analysis.

Spores (3.5 × 10^6^) of the *gfp-hapB* strain were grown in 100 ml of liquid MM for 48 h at 220 rpm and 37°C. ChIP was carried out using GFP-Trap agarose beads (gta-20; ChromoTek) per manufacturer’s instructions (https://www.chromotek.com/downloads/application-notes/). The IgG agarose beads were used as a control. Immunoprecipitated DNA fragments were reverse cross-linked, treated with RNase A (CWBIO, CW0601), and then purified. ChIP-qPCR was performed as previously described with SYBR Premix Ex Taq II (35). The primers for ChIP-qPCR are listed in Table S2. ChIP-qPCR experiments were run in triplicates.

### Data availability.

A processed format of the RNA-seq and proteomics data sets is included in Data Sets S1 and S2, respectively. The raw Illumina sequencing data were uploaded in SRA (https://www.ncbi.nlm.nih.gov/bioproject/PRJNA749483/) at NCBI with accession number PRJNA749483. The mass spectrometry proteomics data have been deposited to the ProteomeXchange Consortium (http://proteomecentral.proteomexchange.org/cgi/GetDataset?ID=PXD027699) via the iProX partner repository (69) with the data set identifier PXD027699.
