# Supplementary figures and images for: The Heterotrimeric Transcription Factor CCAAT-Binding Complex and Ca2+-CrzA Signaling Reversely Regulate the Transition between Fungal Hyphal Growth and Asexual Reproduction
Source: mBio. 2021 Nov 16;12(6):e03007-21. doi: 10.1128/mBio.03007-21 (PMC8593669; doi:10.1128/mBio.03007-21)

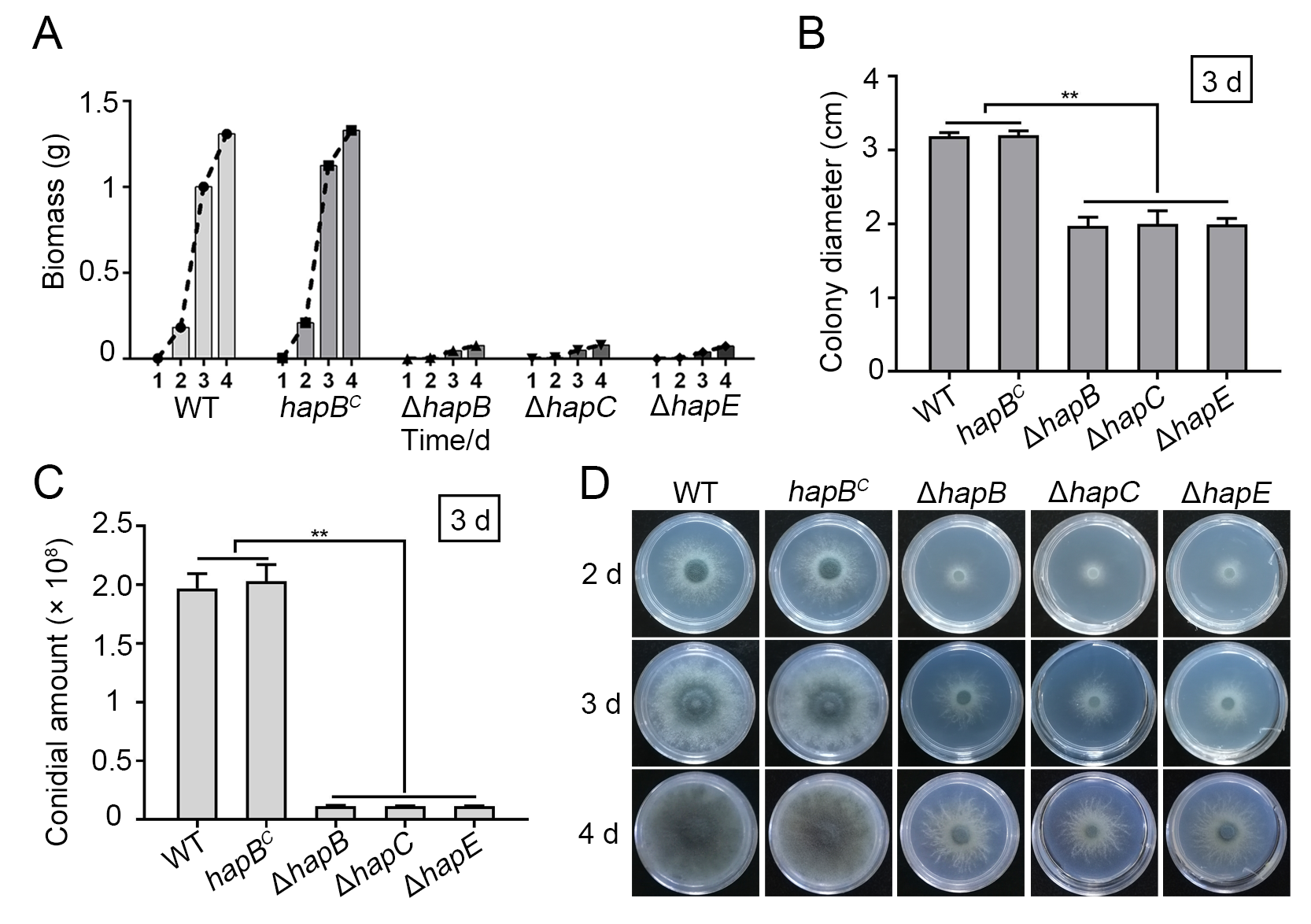

Supplement: FIG S1 [file mbio.03007-21-sf001.tif]

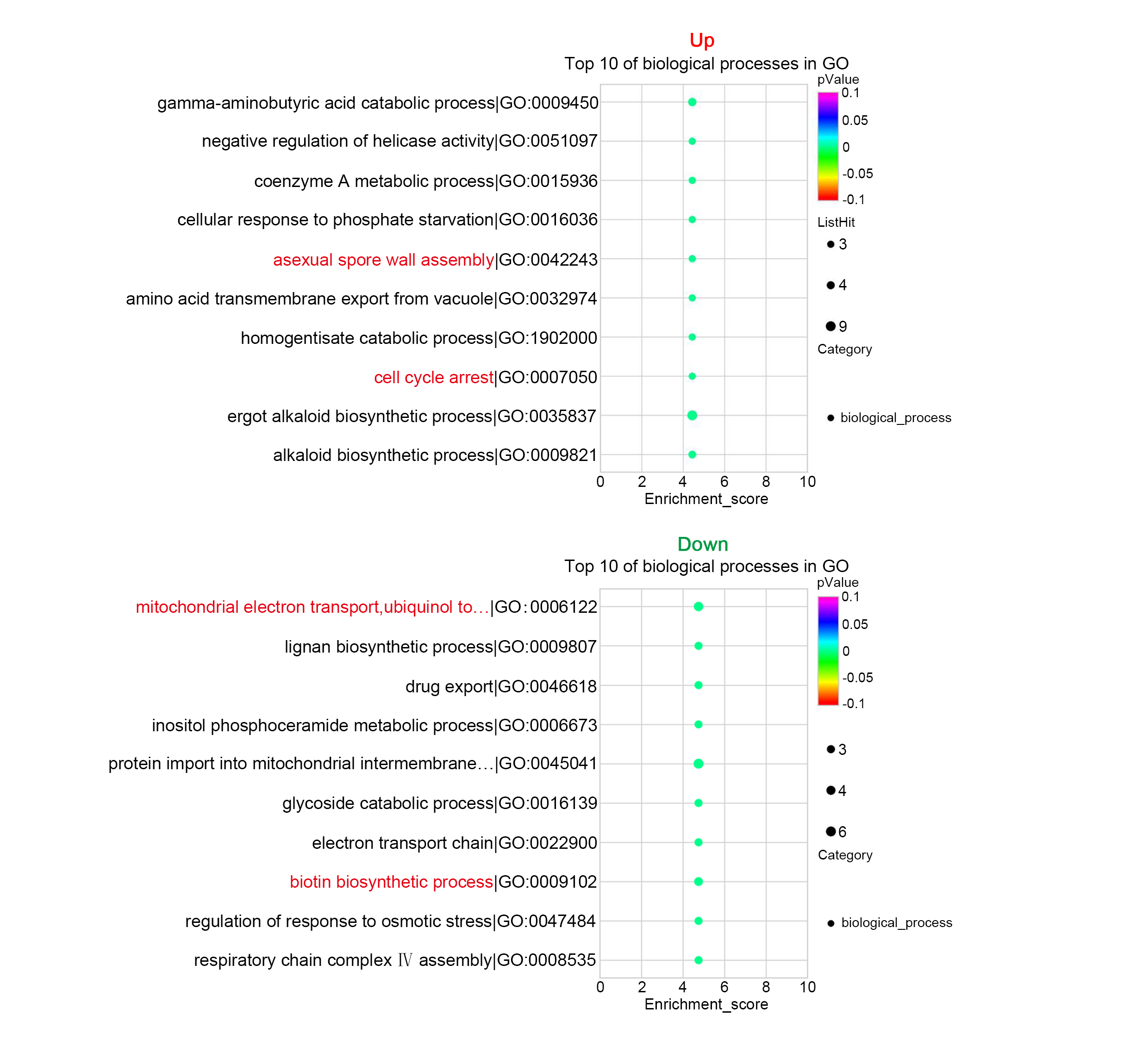

Supplement: FIG S2 [file mbio.03007-21-sf002.tif]

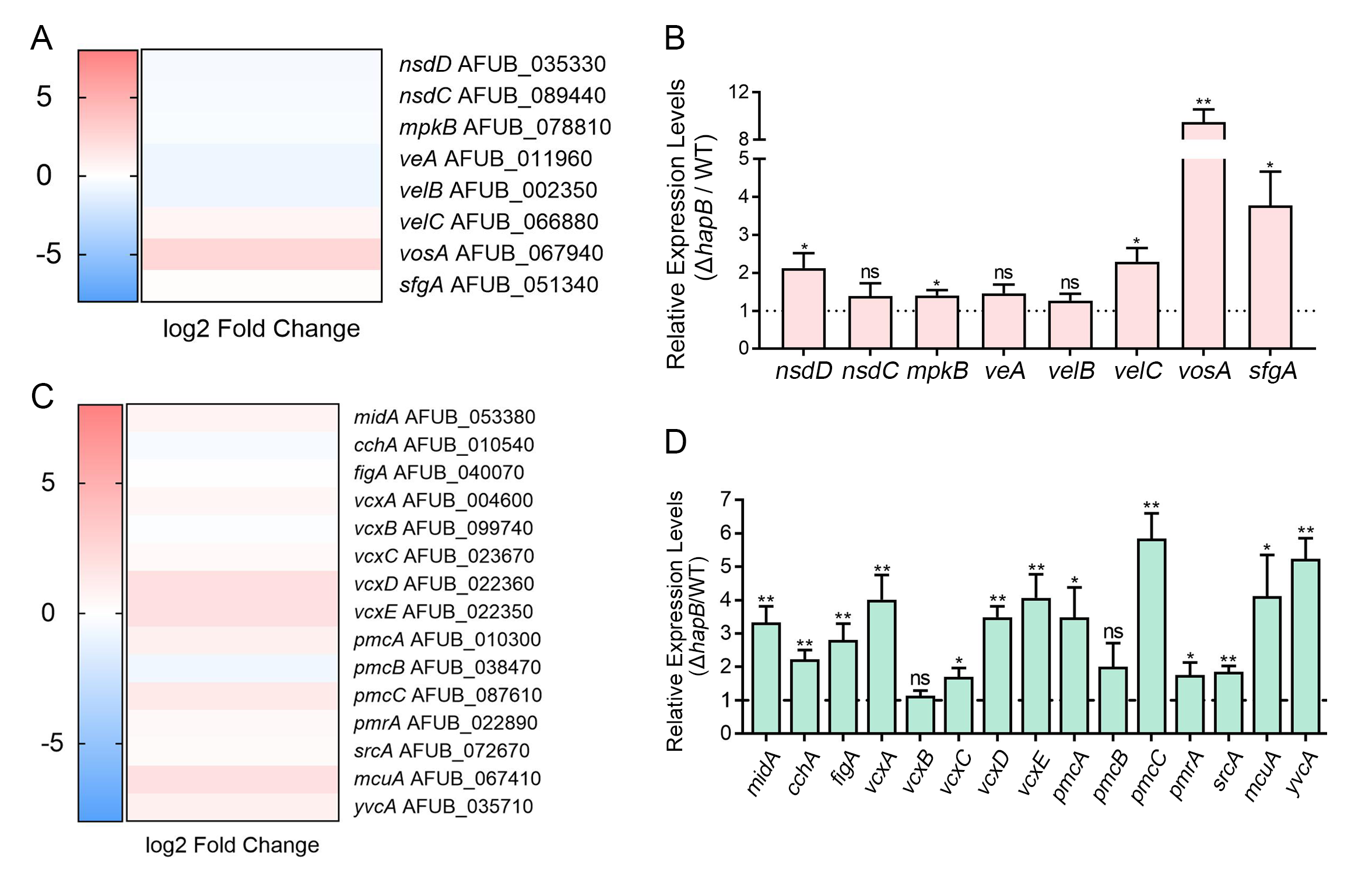

Supplement: FIG S3 [file mbio.03007-21-sf003.tif]

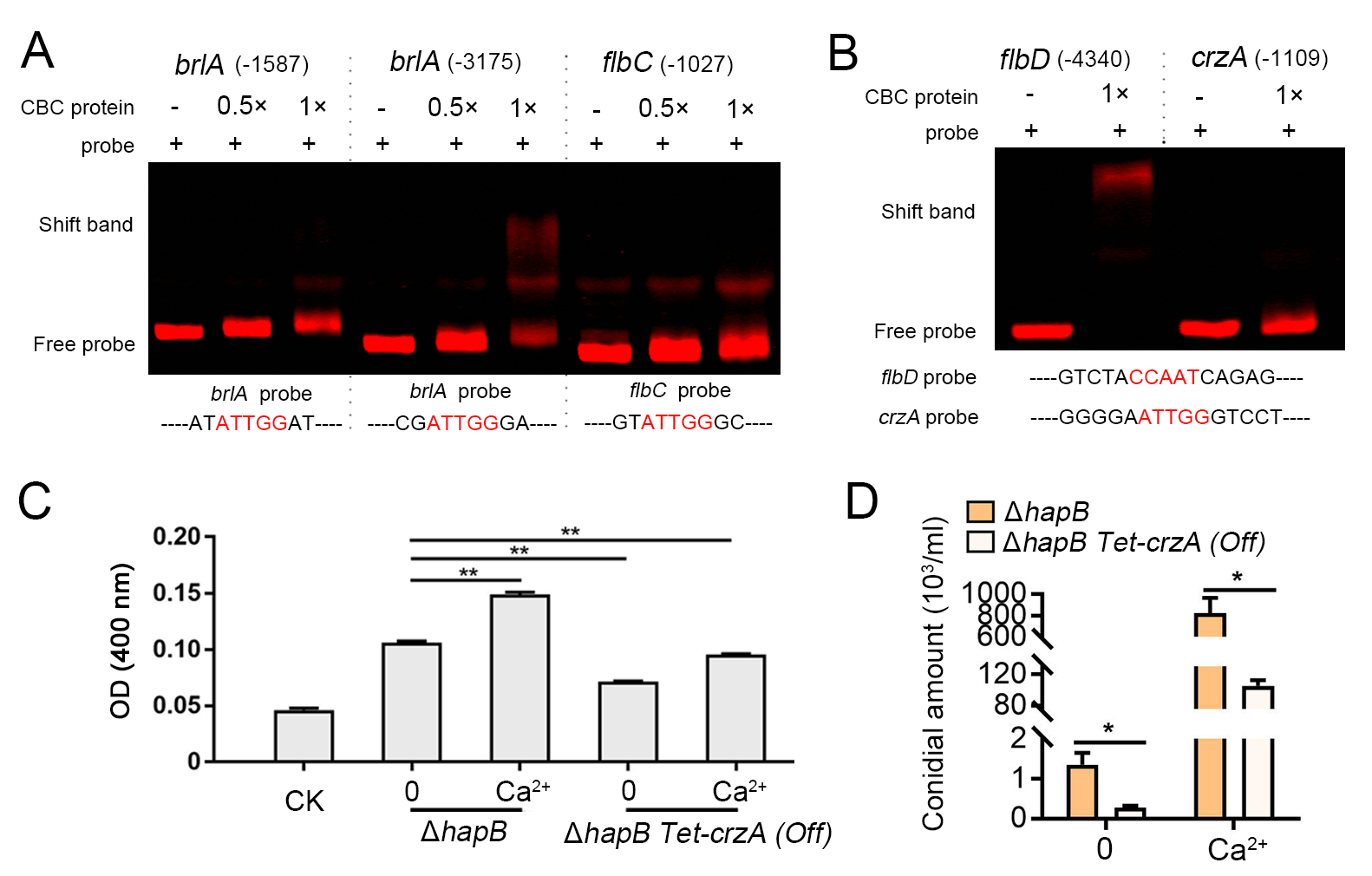

Supplement: FIG S6 [file mbio.03007-21-sf006.tif]

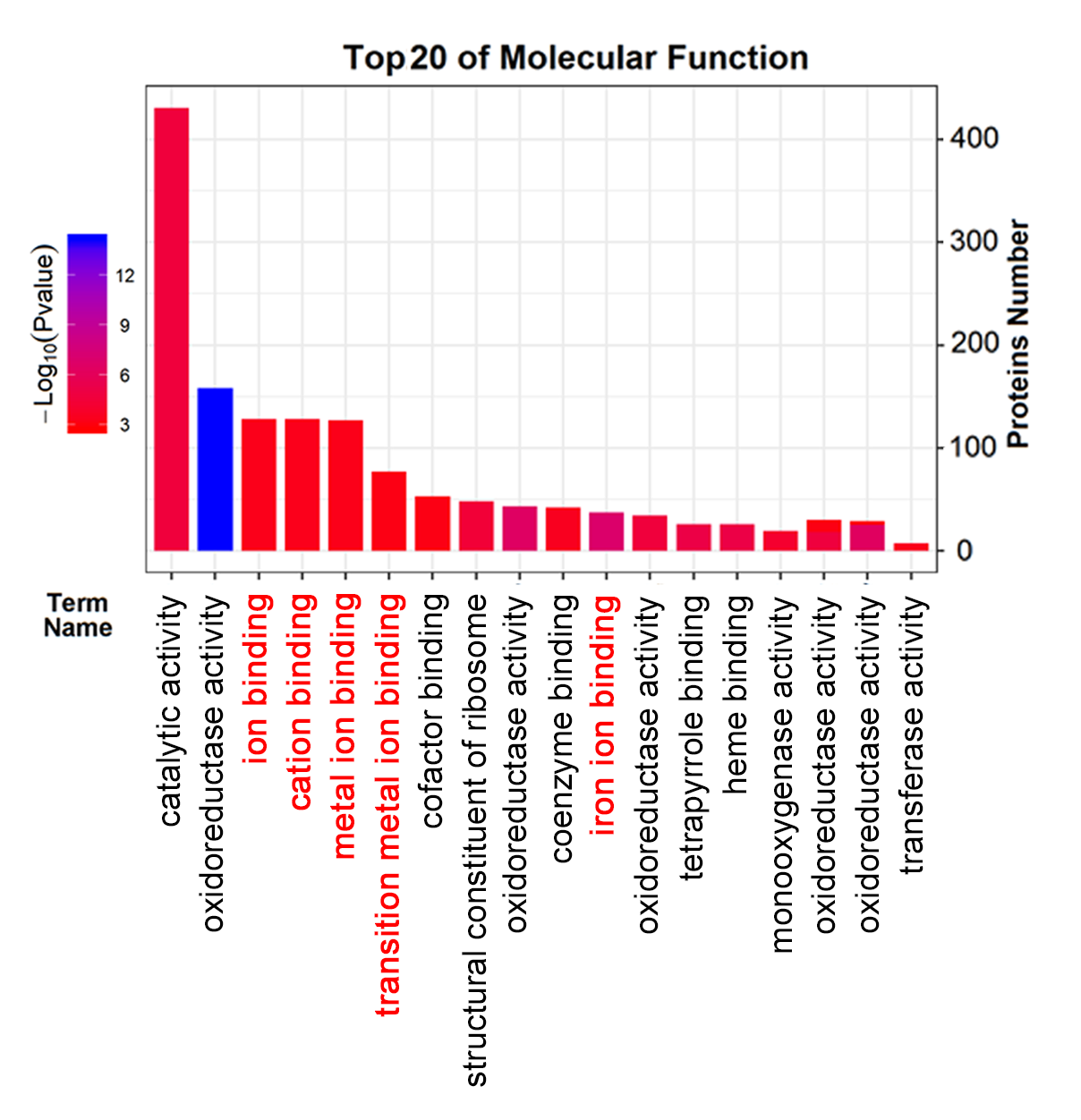

Supplement: FIG S4 [file mbio.03007-21-sf004.tif]

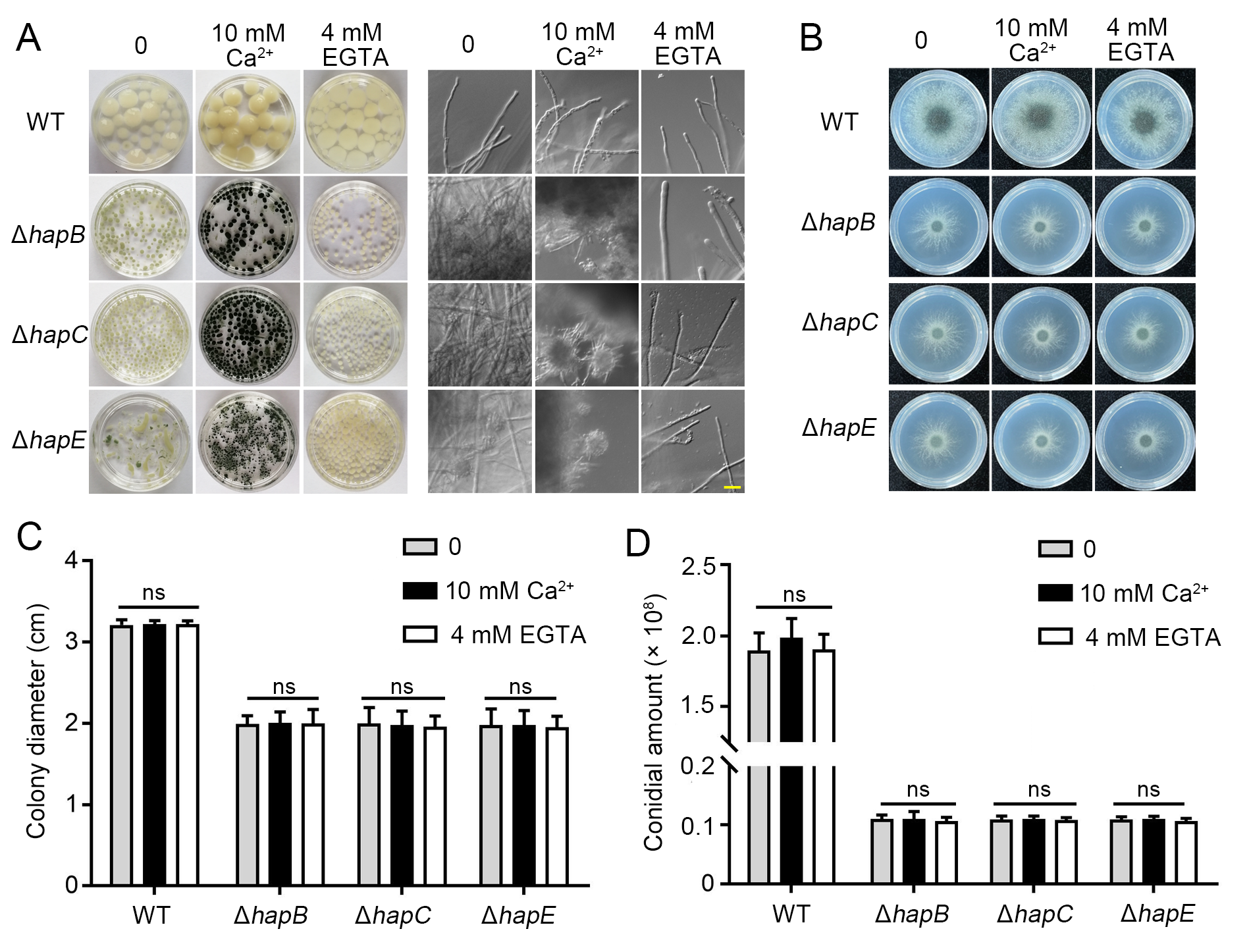

Supplement: FIG S5 [file mbio.03007-21-sf005.tif]
