# Supplementary material for: The Heterotrimeric Transcription Factor CCAAT-Binding Complex and Ca2+-CrzA Signaling Reversely Regulate the Transition between Fungal Hyphal Growth and Asexual Reproduction
Source: mBio. 2021 Nov 16;12(6):e03007-21. doi: 10.1128/mBio.03007-21 (PMC8593669; doi:10.1128/mBio.03007-21)
Supplement: TABLE S1 [file mbio.03007-21-st001.docx]

Table S1. The list of *A. fumigatus* strains used in this study.

| Strains | Genotype and Source | Source­ |
| --- | --- | --- |
| ZC03/WT | Δ*ku80*; *pyrG1*; *AMA1::P_gpdA_::Cas9::pyr4;* from A1160 transformed by plasmid FM-6 | (1) |
| Δ*hapB* | ZC03; Δ*hapB::hph* | This study |
| *hapB^c^* | Δ*ku80*; *pyrG1*; Δ*hapB::hph*; *hapB::pyr4* | This study |
| Δ*hapC* | ZC03; Δ*hapC::hph* | This study |
| Δ*hapE* | ZC03; Δ*hapE::hph* | This study |
| Δ*hapB Tet-flbC* | ZC03; Δ*hapB::hph*; *Tet::flbC::ptrA* | This study |
| Δ*hapB Tet-brlA* | ZC03; Δ*hapB::hph*; *Tet::brlA::ptrA* | This study |
| Δ*hapB Tet-crzA* | ZC03*;* Δ*hapB::hph*; *Tet::crzA::ptrA* | This study |
| WT*^crzA-gfp^* | ZC03; *crzA::gfp::ptrA* | This study |
| Δ*hapB^crzA-gfp^* | ZC03; Δ*hapB::hph*; *crzA::gfp::ptrA* | This study |
| WT^c^ | Δ*ku80*; *pyrG1*; *AMA1::P_gpdA_::Aeq::pyr4* | (2) |
| Δ*hapB^c^* | Δ*ku80*; *pyrG1*; Δ*hapB::hph*; *AMA1::P_gpdA_::Aeq::pyr4* | This study |
| *gfp-hapB* | ZC03; *gfp::hapB*; *hph* | This study |

**Reference**

1. Zhang C, Meng X, Wei X, Lu L. 2016. Highly efficient CRISPR mutagenesis by microhomology-mediated end joining in *Aspergillus fumigatus*. Fungal Genet Biol 86:47-57.

2. Zhang C, Ren Y, Gu H, Gao L, Zhang Y, Lu L. 2021. Calcineurin-mediated intracellular organelle calcium homeostasis is required for the survival of fungal pathogens upon extracellular calcium stimuli. Virulence 12:1091-1110.
