# Supplementary material for: The Heterotrimeric Transcription Factor CCAAT-Binding Complex and Ca2+-CrzA Signaling Reversely Regulate the Transition between Fungal Hyphal Growth and Asexual Reproduction
Source: mBio. 2021 Nov 16;12(6):e03007-21. doi: 10.1128/mBio.03007-21 (PMC8593669; doi:10.1128/mBio.03007-21)
Supplement: TABLE S2 [file mbio.03007-21-st002.docx]

Table S2. Primers used in this study.

| Name | Sequence | Intention |
| --- | --- | --- |
| T7-hapB-sgRNA1-F | TAATACGACTCACTATAGGGAGGGTATTCCATCGTGTGTTTTAGAGCTAGAAATAGC | for the DNA template of hapB-sgRNA1(RNA); for deleting *hapB*；for *gfp-hapB* |
| hapB-deletion-hph-F | CCAAGGTGACGGAACTTTCCGTCGCGTCTCAACAGAATTCCCTTGTATCTCTACACAC | for the repair template of deleting *hapB* |
| hapB-deletion-hph-R | CTCATACGACGCTGTTGCATCTGGGCATTTTGGGTCGAGTGGAGATGTGGAGTGGG | for the repair template of deleting *hapB* |
| hapB-N-gfp-F | AAGGTGACGGAACTTTCCGTCGCGTCTCCAACACGATGAGTAAAGGAGAAGAACTTTTC | for the repair template of constructing *gfp-hapB* |
| hapB-N-gfp-R | CTGGCCATGAGGCTGTTGATATTGTGGAGGGTATTCCTGGATCTCGGAGATTTTGTATA | for the repair template of constructing *gfp-hapB* |
| hapB-seq-F | ACTCGAGCTGTTACTGGGTTAAG | diagnostic primer for Δ*hapB* and *gfp-hapB* |
| hapB-seq-R | ATGAGCCGATCGACAAGAGGGTA | diagnostic primer for Δ*hapB* and *gfp-hapB* |
| NotI-hapB-F | AAGGGCAATTCGCGGCCTCAGCACGCGTCAATCATATCT | for revertant of *hapB* mutant |
| NotI-hapB-R | CGAATTGAATTTAGCGGCCCGTGGTCGAGGTTGCAGTGTA | for revertant of *hapB* mutant |
| T7-hapC-sgRNA-F | TAATACGACTCACTATAGGGTGCTACCCTTGTTTCGGTTTTAGAGCTAGAAATAGC | for the DNA template of hapC-sgRNA (RNA); for deleting *hapC* |
| hapC-deletion-hph-F | AGTATCCGCTGCGGGTATACCTATAGCTAAAAATCTCGAGTGGAGATGTGGAGTGG | for the repair template of deleting *hapC* |
| hapC-deletion-hph-R | TCGAGACTCTGTTCCATGGCAGACTCGACCCCGAGAATTCCCTTGTATCTCTACACA | for the repair template of deleting *hapC* |
| hapC-seq-F | CCGTCAGGATTATACCGTCAGGG | diagnostic primer for Δ*hapC* |
| hapC-seq-R | CTGGTCCTGTTTACCTCTCTGTCG | diagnostic primer for Δ*hapC* |
| T7-hapE-sgRNA-F | TAATACGACTCACTATAGGGCGGTGCGACGCGGTAGGGTTTTAGAGCTAGAAATAGC | for the DNA template of hapE-sgRNA (RNA); for deleting *hapE* |
| hapE-deletion-hph-F | TTTTTAATTTATTATATTCACAGGTCCCGCAACCGCCTTCGAGTGGAGATGTGGAGTGG | for the repair template of deleting *hapE* |
| hapE-deletion-hph-R | AAAGGAGCATGGTTAGCTTAATCGCGTACCGACATGAATTCCCTTGTATCTCTACACA | for the repair template of deleting *hapE* |
| hapE-seq-F | CGACATCTTTGTATCAATCCAAG | diagnostic primer for Δ*hapE* |
| hapE-seq-R | CCGGAGTCGATGTTAAGCATTCTG | diagnostic primer for Δ*hapE* |
| sgRNA-R | AAAAAAGCACCGACTCGGTGCC | the DNA template of sgRNA |
| T7-tet-flbC-F | TAATACGACTCACTATAGGGTCATTGTCCTGCCTTAGTTTTAGAGCTAGAAATAGCA | for the DNA template of flbC-sgRNA (RNA); for constructing *Tet-flbC* |
| ptrA-tet-flbC-F | GCAGATTATCACCCTTCTGGTCATTGTCCTGCCGCCTAGATGGCCTCTTGCATC | for constructing *Tet-flbC* |
| Tet-flbC-R | ATTGGCGGTTCTGGTTCTCAATGACCATCGTCATGGTGATGTCTGCTCAAGCGG | for constructing *Tet-flbC* |
| flbC-seq-F | CCATCACTGCTCATGCTGAG | diagnostic primer for *Tet-flbC* |
| flbC-seq-R | GAGGAGTCGAGTGGGAAGAC | diagnostic primer for *Tet-flbC* |
| T7-tet-brlA-F | TAATACGACTCACTATAGGGCCTCGCAGCCTGATACGTTTTAGAGCTAGAAATAGCA | for the DNA template of brlA-sgRNA (RNA); for constructing *Tet-brlA* |
| ptrA-tet-brlA-F | GCAACACTGTCTCTCGTTGGCCTCGCAGCCTGAGCCTAGATGGCCTCTTGCATC | for constructing *Tet-brlA* |
| Tet-brlA-R | CAGGCGATCAGACATATTACCCTGGGATCTCATGGTGATGTCTGCTCAAGCGG | for constructing *Tet-brlA* |
| T7-tet-crzA-F | TAATACGACTCACTATAGGGATCAACGAGCGCAGAGGTTTTAGAGCTAGAAATAGCA | for the DNA template of crzA-sgRNA; for constructing *Tet-crzA* |
| ptrA-tet-crzA-F | CTTCTCTCCGCACCCGCATTGGTTATCCTCTCGCCTAGATGGCCTCTTGCATC | for constructing *Tet-crzA* |
| Tet-crzA-R | GCCCCAACTCAGGGAACATCTCCTGTGAAGCCATGGTGATGTCTGCTCAAGCGG | for constructing *Tet-crzA* |
| crzA-seq-F | CGGAAACCTCGGATGGAGCTC | diagnostic primer for *TeT-crzA* |
| crzA-seq-R | ACGACCATCTTCATTGCTAAGC | diagnostic primer for *TeT-crzA* |
| T7-crzA-gfp-F | TAATACGACTCACTATAGGGATGACAGTAGACAGACGTTTTAGAGCTAGAAATAGCA | for the DNA template of crzA-sgRNA1; for constructing *crzA-gfp* |
| crzA-gfp-F | TGCTGGGTATTCTGCTGCCGGTAACTTCTATGGAGCTGGTGCAGGCGCTGG | for constructing *crzA-gfp* |
| ptrA-crzA-gfp-R | GGTGAACGTGAGAACGGGGATGACAGTAGACACATGGCAGACACTGAAGCAAC | for constructing *crzA-gfp* |
| crzA-gfp-seq-F | CTCCACCTTCGTCCACACGCTC | diagnostic primer for *crzA-gfp* |
| crzA-gfp-seq-R | GAGATTGAATGCGAAGCCTATC | diagnostic primer for *crzA-gfp* |
| tubA-RT-F | TGACTGCCTCCAGGGCTTCC | RT-qPCR primer |
| tubA-RT-R | GCGTTGTAAGGCTCAACGAC | RT-qPCR primer |
| pksP-RT-F | GAAGATGCAGCCGTGGAAGG | RT-qPCR primer |
| pksP-RT-R | GGGAGGAGCTTTCTTCTCCC | RT-qPCR primer |
| Ayg1-RT-F | GCTTGTAGAGACGGGCATCG | RT-qPCR primer |
| Ayg1-RT-R | CTGCTCCAACCACTCGTAGG | RT-qPCR primer |
| Arp2-RT-F | GCTGGAGGACTTCAACGAGG | RT-qPCR primer |
| Arp2-RT-R | CACCCCTTGCGCCCAAAATC | RT-qPCR primer |
| Arp1-RT-F | CCAATCTGACACTCGAGTTC | RT-qPCR primer |
| Arp1-RT-R | CCATGTCAGAAATCATGGCC | RT-qPCR primer |
| Arb1-RT-F | CAGATGCATCTGGCTCACCC | RT-qPCR primer |
| Arb1-RT-R | GAAGGCAATGGAGGAGACCG | RT-qPCR primer |
| Arb2-RT-F | GAAGGCAATGGAGGAGACCG | RT-qPCR primer |
| Arb2-RT-R | GGGTGATGTTCCCAGTTGCC | RT-qPCR primer |
| Abr1-RT-F | CCGGAAACACCAAGGATTTG | RT-qPCR primer |
| Abr1-RT-R | CTCGTCCGAATCATTACTGG | RT-qPCR primer |
| FahA-RT-F | CGGTACCATTTCTGGCAAGG | RT-qPCR primer |
| FahA-RT-R | GAATTGTGCCAACGCAGTCG | RT-qPCR primer |
| HmgA-RT-F | GTTCATGGGGCTGATCTGCG | RT-qPCR primer |
| HmgA-RT-F | CCCATTCGGATACGCCAACC | RT-qPCR primer |
| HppD-RT-F | GTGCGGCTGAAGAAAGCTGG | RT-qPCR primer |
| HppD-RT-R | CCCGCTCAATGGCCTCAAAG | RT-qPCR primer |
| MaiA-RT-F | CGAAGAGTGCGGGGCTTTTC | RT-qPCR primer |
| MaiA-RT-R | CTCACTCGAAACTCCTCCGG | RT-qPCR primer |
| HmgX-RT-F | CGGGAATGAGATGGTAGGGG | RT-qPCR primer |
| HmgX-RT-R | CTTCCTTCCTCAACGCCTCC | RT-qPCR primer |
| RodA-RT-F | GAGGGCATCCTTGCTGGTAC | RT-qPCR primer |
| RodA-RT-R | GGGCAATGCAAGGAAGACCC | RT-qPCR primer |
| RodB-RT-F | CCTTCAACTACGGCCTCCTG | RT-qPCR primer |
| RodB-RT-R | GGCAATGTTGATGAGGCCGC | RT-qPCR primer |
| fluG-RT-F | CTGGGAACTCAAGTCCCTCG | RT-qPCR primer |
| fluG-RT-R | CGAGCGCATCAAGACTCTGC | RT-qPCR primer |
| flbA-RT-F | CAACCGCGGTATGCCAAAGG | RT-qPCR primer |
| flbA-RT-R | CTAAGCTGTTTCGCAGGGCG | RT-qPCR primer |
| flbB-RT-F | GGCTGTTGGGGGTGTATTCG | RT-qPCR primer |
| flbB-RT-R | GCTCTGGAGTTGTGTTGGCG | RT-qPCR primer |
| flbC-RT-F | CTCCCGGCCCACAATCTATG | RT-qPCR primer |
| flbC-RT-R | GTGGACCTTCTTGTGCCGAC | RT-qPCR primer |
| flbD-RT-F | CGAGGATGGATACGTGAGCC | RT-qPCR primer |
| flbD-RT-F | GAGACCCATTCGCGCATCTC | RT-qPCR primer |
| brlA-RT-F | GGGCCATACGGAGTCGATTG | RT-qPCR primer |
| brlA-RT-R | GGCGAGTGCGTCTTGAAGGT | RT-qPCR primer |
| AbaA-RT-F | GACTGGCAGCCCGAGTGTATT | RT-qPCR primer |
| AbaA-RT-R | GTCATCACGACCACTCATCCC | RT-qPCR primer |
| WetA-RT-F | CCCATCAGTCACCGCAACC | RT-qPCR primer |
| WetA-RT-R | GGGAGGAGTGGACGGTGAT | RT-qPCR primer |
| CrzA-RT-F | GACCTTCAAAACCGGCCAGC | RT-qPCR primer |
| CrzA-RT-R | CGCGTGAGCTTGGATTGCTC | RT-qPCR primer |
| NsdD-RT-F | GAACCAGGAGCATGCCTTGG | RT-qPCR primer |
| NsdD-RT-R | CGCCATTCTGGAGTTTCGGC | RT-qPCR primer |
| NsdC-RT-F | CACAGCAGGCAATGGCACTC | RT-qPCR primer |
| NsdC-RT-R | CTGTGCAGTGCTACCGGAAC | RT-qPCR primer |
| MpkB-RT-F | TGTCGTTTGCTCGGCTATCC | RT-qPCR primer |
| MpkB-RT-R | CTGAATCAAATACACCTCGTTG | RT-qPCR primer |
| VeA-RT-F | CATGTAACTTCACTCCCGCC | RT-qPCR primer |
| VeA-RT-R | GATCCATATCAGGCCGCATG | RT-qPCR primer |
| VelB-RT-F | GCCAGCGCCTTTAGACTCAC | RT-qPCR primer |
| VelB-RT-R | CTTGGAACGGCTCGGAGAAG | RT-qPCR primer |
| VelC-RT-F | GCGGATTCCATAGGCCAAAC | RT-qPCR primer |
| VelC-RT-R | GCTGAGCTGCGATCAGAATG | RT-qPCR primer |
| VosA-RT-F | CAAATGCAGGATCCAGCGGC | RT-qPCR primer |
| VosA-RT-R | GATGCACCGTTGGTCCCTTG | RT-qPCR primer |
| SfgA-RT-F | CACGGTTGGGTCTCGATTGC | RT-qPCR primer |
| SfgA-RT-R | GATCATCCTTCTCGTCCCGG | RT-qPCR primer |
| CchA-RT-F | AGCACAAGAATGCACGACTCG | RT-qPCR primer |
| CchA-RT-R | GATTGTTGTACATCCTCCACGC | RT-qPCR primer |
| MidA-RT-F | GTGAGTTGCAACGACTGTGC | RT-qPCR primer |
| MidA-RT-R | GATGTCTTGGCACGGTAAGA | RT-qPCR primer |
| FigA-RT-F | CACGGCATTGGCTGAGATTG | RT-qPCR primer |
| FigA-RT-R | CGCGAGGGAAATGGCTTAAC | RT-qPCR primer |
| PmrA-RT-F | CCTTTGGATCAATATCCTCATG | RT-qPCR primer |
| PmrA-RT-R | AGCAGGTGAACGTCATGGTCG | RT-qPCR primer |
| McuA-RT-F | GCCGTCGAGGTTGTTTAAAC | RT-qPCR primer |
| McuA-RT-R | CAAGCTGAAGCGCGATGAAT | RT-qPCR primer |
| SrcA-RT-F | CAATGACCCTCCATTTCGCC | RT-qPCR primer |
| SrcA-RT-R | GCCACTCCATTCTGCTGTTC | RT-qPCR primer |
| PmcA-RT-F | CGATCTCGCTCTAACTCTGC | RT-qPCR primer |
| PmcA-RT-R | TAATCTCCGACGAGACGCTC | RT-qPCR primer |
| PmcB-RT-F | ATGTGACTCGCCTGGACGCTG | RT-qPCR primer |
| PmcB-RT-R | CAGGCCACATCATAGACGAG | RT-qPCR primer |
| PmcC-RT-F | CTCTGGCCTTAGCAACCGAC | RT-qPCR primer |
| PmcC-RT-R | TGAAGATGACGGTGTCGAGC | RT-qPCR primer |
| YvcA-RT-F | GCGATCTTCGCTCTGAAGAC | RT-qPCR primer |
| YvcA-RT-R | ATCCTCTTCTCCCTGACGAC | RT-qPCR primer |
| VcxA-RT-F | GATGACTCTCAACTTCCACATC | RT-qPCR primer |
| VcxA-RT-R | GCATCGCCAGTAGGATCATC | RT-qPCR primer |
| VcxB-RT-F | GTCATGTTACTGGTGTCAACTGC | RT-qPCR primer |
| VcxB-RT-R | TGTCTTTGTCCATGCACCAGC | RT-qPCR primer |
| VcxC-RT-F | CACCAAGCTCACTCTGACC | RT-qPCR primer |
| VcxC-RT-R | GATTTGATTGTCCCAGGCTTCGTC | RT-qPCR primer |
| VcxD-RT-F | GAACCCATCAACGTGTCCAAC | RT-qPCR primer |
| VcxD-RT-R | GTAATTCAGTGCAATTCCAACTGG | RT-qPCR primer |
| VcxE-RT-F | GTTGGTTCCATTGACGCTCTTAC | RT-qPCR primer |
| VcxE-RT-R | CCATCGGCAATGAGATAGTTCAC | RT-qPCR primer |
| CrzA-RT-F | CGCTGCTAGATGAGGAGTCG | RT-qPCR primer |
| CrzA-RT-R | TGGTCCCACTGCAAGGTCTG | RT-qPCR primer |
| Pet30A-F | GAATTCGAGCTCCGTCGACACCAGCTTGCGGCCGCACTCGAG | Linearization of Pet30A |
| Pet30A-R | CATATGTATATCTCCTTCTTAAAGTTAAAC | Linearization of Pet30A |
| EmsA-hapB-F | GAAGGAGATATACATATGATGGAATACCCTCCACAATA  TCAAC | Amplification of *hapB* cDNA |
| EmsA-hapB-R | GTCGACGGAGCTCGAATTCACCATCTTCATCGGTTGGTTC | Amplification of *hapB* cDNA |
| EmsA-hapC-F | GAAGGAGATATACATATGATGTCGGCCTCTCCCTCGAAAG | Amplification of *hapC* cDNA |
| EmsA-hapC-R | GTCGACGGAGCTCGAATTCGTACGAGTCGCCTCCTGCTC | Amplification of *hapC* cDNA |
| EmsA-hapE-F | GAAGGAGATATACATATGATGGAACAGTCTTCGCAGAGCAC | Amplification of *hapE* cDNA |
| EmsA-hapE-R | GTCGACGGAGCTCGAATTCAGAAGAGTTTTGGCATACCTGC | Amplification of *hapE* cDNA |
| FluG-probe-F | AGATGCAGCTAGCACGCGATCGTCTAGTTCTCCTATTC | for *fluG* probe (-335) and *fluG* mutant probe (-335) |
| FluG-Joint-P-R | GTTTCACATCTCGTGGACGAACAGCCGATCCTTCCAAG | for *fluG* mutant probe (-335) |
| FluG-Joint-P-F | CGTCCACGAGATGTGAAAC | for *fluG* mutant probe (-335) |
| FluG-probe-R | AGATGCAGCTAGCACGGGCAGAGCAGATGCTTACTC | for *fluG* probe (-335) and *fluG* mutant probe (-335) |
| EMSA-fluG-F | AGATGCAGCTAGCACGGTTGCCTGTCGCTTCCAATGACGAGTTGTGTAGTTAGTTATC | for *fluG* probe (-788) |
| EMSA-fluG-MF | AGATGCAGCTAGCACGGTTGCCTGTCGCTTCATATGACGAGTTGTGTAGTTAGTTATC | for *fluG* mutant probe (-788) |
| EMSA-fluG-R | AGATGCAGCTAGCACGTTGCTGCGCTCGGTAGCTCG | for *fluG* probe (-788) and *fluG* mutant probe (-788) |
| EMSA-flbD-1-F | AGATGCAGCTAGCACGTTGACTTACAATCGACACCAATCAACTGCTGACTCCGTTTTG | for *flbD* probe (-247) |
| EMSA-flbD-1-MF | AGATGCAGCTAGCACGTTGACTTACAATCGACACAAAGCAACTGCTGACTCCGTTTTG | for *flbD* mutant probe (-247) |
| EMSA-flbD--1-R | AGATGCAGCTAGCACGGCGTTGCAGACAGCGGAAGG | for *flbD* probe (-247) and *flbD* mutant probe (-247) |
| EMSA-flbD-2-F | AGATGCAGCTAGCACGTACCTTAAAATCAGTCTACCAATCAGAGACTTTGTTTGGCGA | for *flbD* mutant probe (-4340) |
| EMSA-flbD-2-MF | AGATGCAGCTAGCACGTACCTTAAAATCAGTCTACAGTTCAGAGACTTTGTTTGGCGA | for *flbD* mutant probe (-4340) |
| EMSA-flbD-2-R | AGATGCAGCTAGCACGATCCGAGGAATAGAAGCACG | for *flbD* probe (-4340) and *flbD* mutant probe (-4340) |
| EMSA-brlA-1-F | AGATGCAGCTAGCACGCAACGGAACTGGCTAATATTGGTTGACTTGTCGCGTGCACTTG | for *brlA* probe (-4421) |
| EMSA-brlA-1-MF | AGATGCAGCTAGCACGCAACGGAACTGGCTAATTATGTTTGACTTGTCGCGTGCACTTG | for *brlA* mutant probe (-4421) |
| EMSA-brlA-1-R | AGATGCAGCTAGCACGCTGCGTCGGTAATGAAGAAC | for *brlA* probe (-4421) and *brlA* mutant probe (-4421) |
| EMSA-brlA-2-F | AGATGCAGCTAGCACGCAGTGTCGCACAGACCGCGATTGGGATTGTCAGCAAACACTAG | for *brlA* probe (-3175) |
| EMSA-brlA-2-MF | AGATGCAGCTAGCACGCAGTGTCGCACAGACCGCGTGAGTGATTGTCAGCAAACACTAG | for *brlA* mutant probe (-3175) |
| EMSA-brlA-2-R | AGATGCAGCTAGCACGCTGAGACAGATAATATTAGGC | for *brlA* probe (-3175) and *brlA* mutant probe (-3175) |
| EMSA-brlA-3-F | AGATGCAGCTAGCACGCCAAACATTCCAGATGGCTC | for *brlA* probe (-1587) |
| EMSA-brlA-3-R | AGATGCAGCTAGCACGCCCTGTGCATGATTGATTTGGATCCAAT | for *brlA* probe (-1587) |
| EMSA-flbC-1-F | AGATGCAGCTAGCACGGATGCTGCTGTTGCTCCCAATCATGGTGATGATGACATGAG | for *flbC* probe (-3268) |
| EMSA-flbC-1-MF | AGATGCAGCTAGCACGGATGCTGCTGTTGCTCCTGATCATGGTGATGATGACATGAG | for *flbC* mutant probe (-3268) |
| EMSA-flbC-1-R | AGATGCAGCTAGCACGGAGAAAAGCATCGCATGTGC | for *flbC* probe (-3268) and *flbC* mutant probe (-3268) |
| EMSA-flbC-2-F | AGATGCAGCTAGCACGTCTTTAAAACTGGTATGGTATTGGGCTTTGTCGCATTATTCG | for *flbC* probe (-1027) |
| EMSA-flbC-2-R | AGATGCAGCTAGCACGGCTCTCCTAATAAAACTGCTTC | for *flbC* probe (-1027) |
| EMSA-crzA-1-F | AGATGCAGCTAGCACGCTCCTTCTCTCCGCACCCGCATTGGTTATCCTCTCTGCGCT | for *crzA* probe (-207) |
| EMSA-crzA-1-MF | AGATGCAGCTAGCACGCTCCTTCTCTCCGCACCCGCAGTAGTTATCCTCTCTGCGCT | for *crzA* mutant probe (-207) |
| EMSA-crzA-1-R | AGATGCAGCTAGCACGCCGTAGTATGTCTGTATAGG | for *crzA* probe (-207) and *crzA* mutant probe (-207) |
| EMSA-crzA-2-F | AGATGCAGCTAGCACGAGCCGATCAATCATGGGGAATTGGGTCCTAATTCATGTGGGGA | for *crzA* probe (-1109) |
| EMSA-crzA-2-MF | AGATGCAGCTAGCACGAGCCGATCAATCATGGGGAATCAGGTCCTAATTCATGTGGGGA | for *crzA* mutant probe (-1109) |
| EMSA-crzA-2-R | AGATGCAGCTAGCACGTCCATCAACCATCCCGGTGAC | for *crzA* probe (-1109) and *crzA* mutant probe (-1109) |
| brlA-4288-4445-F | ATTGGTTGACTTGTCGCGTG | for ChIP-qPCR |
| brlA-4288-4445-R | GCAGGCCTCTTATTCTCGGT | for ChIP-qPCR |
| flbC-3164-3288-Primer-F | GCTGTTGCTCCCAATCAT | for ChIP-qPCR |
| flbC-3164-3288-Primer-R | CCGTCTTTACCACAAACCT | for ChIP-qPCR |
| flbD-4265-4359-Primer-F | ATCAGTCTACCAATCAGAGA | for ChIP-qPCR |
| flbD-4265-4359-Primer-R | GGAATAGAAGCACGAACC | for ChIP-qPCR |
| fluG-251-358-Primer-F | TTGGAAGGATCGGCTGTTCC | for ChIP-qPCR |
| fluG-251-358-Primer-R | GCAGATGCTTACTCGATTAGGA | for ChIP-qPCR |
| CrzA-101-230-Primer-F | GCACCCGCATTGGTTATCCT | for ChIP-qPCR |
| CrzA-101-230-Primer-R | GATAGAGAAGCGCCGCCGTA | for ChIP-qPCR |
| brlA-3089-3199-Primer-F | CACAGACCGCGATTGGGATT | for ChIP-qPCR |
| brlA-3089-3199-Primer-R | TGAGACAGATAATATTAGGCACATCA | for ChIP-qPCR |
| fluG-669-814-Primer-F | TCCAATGACGAGTTGTGTA | for ChIP-qPCR |
| fluG-669-814-Primer-R | AATCTTCCACGAGCTGTC | for ChIP-qPCR |
| flbD-200-268-Primer-F | GACTTACAATCGACACCAATC | for ChIP-qPCR |
| flbD-200-268-Primer-R | AGACAGCGGAAGGAGACT | for ChIP-qPCR |
| AFUB_041590  -Primer-F | TGAGCTCAAACTGACGGATG | for ChIP-qPCR |
| AFUB_041590  -Primer-R | CTCTCGGAAATGGAGGTGAA | for ChIP-qPCR |
